# Cyclodipeptides: From Their Green Synthesis to Anti-Age Activity

**DOI:** 10.3390/biomedicines10102342

**Published:** 2022-09-20

**Authors:** Veronica Mosetti, Beatrice Rosetti, Giovanni Pierri, Ottavia Bellotto, Simone Adorinni, Antonella Bandiera, Gianpiero Adami, Consiglia Tedesco, Matteo Crosera, Greta Camilla Magnano, Silvia Marchesan

**Affiliations:** 1 Chemical and Pharmaceutical Sciences Department, University of Trieste, 34127 Trieste, Italy; 2 Department of Chemistry and Biology “A. Zambelli”, University of Salerno, 84084 Fisciano, Italy; 3 Life Sciences Department, University of Trieste, 34127 Trieste, Italy

**Keywords:** cyclodipeptides, diketopiperazines, anti-age, cosmetics, peptides, green synthesis, microwave, UV-damage, Franz cell, skin absorption

## Abstract

Cyclodipeptides (CDPs) or diketopiperazines (DKPs) are often found in nature and in foodstuff and beverages and have attracted great interest for their bioactivities, biocompatibility, and biodegradability. In the laboratory, they can be prepared by green procedures, such as microwave-assisted cyclization of linear dipeptides in water, as performed in this study. In particular, five CDPs were prepared and characterized by a variety of methods, including NMR and ESI-MS spectroscopies and single-crystal X-ray diffraction (XRD), and their cytocompatibility and anti-aging activity was tested in vitro, as well as their ability to penetrate the different layers of the skin. Although their mechanism of action remains to be elucidated, this proof-of-concept study lays the basis for their future use in anti-age cosmetic applications.

## 1. Introduction

Cyclodipeptides (CDPs) or 2,5-diketopiperazines (DKPs) are naturally occurring biomolecules that have increasingly attracted researchers’ attention for a variety of reasons, as evident from the many recent reviews that cover aspects ranging from their sustainable production via enzymatic catalysis [1] to all the possible methods for their preparation and self-assembly into gels [2] and their biological activities [3,4], including their function as anti-cancer agents [5]. First of all, compared to linear dipeptide analogues, they lack the charged termini, thus being more lipophilic with a fast membrane absorption in the digestive tract, thanks to their high permeability [6]. Secondly, they have less conformational freedom and a unique structural rigidity [7], which confers them with a higher resistance against enzymatic degradation, especially for those containing proline [8]. The presence of the six-membered piperazine ring with sidechains that are oriented in a spatially defined manner allows the accurate prediction of their conformation too. Thanks to their rigid backbone, they can mimic secondary structural motifs, especially β-turns, which are present in many bioactive compounds [9]. The spectrum of their biological activities indeed encompasses antimicrobial, antitumoral, and neuroprotective effects [10].

Furthermore, they have been recently proposed as anti-age compounds, in light of their established activity to reduce advanced glycation end products (AGEs) that accumulate in fibroblasts during ageing [11]. AGEs accumulation in the skin is known to have negative effects from an aesthetic point of view, such as the formation of wrinkles, yellowish complexion, and brown spots [12].

In addition, the naturally occurring, non-enzymatic glycation of proteins has been linked to several age-related pathologies. For instance, this post-translational modification was shown to affect amyloid formation [13] and was associated with diabetes [14], non-alcoholic fatty liver disease [15], thyroid cancer progression [16], coronary artery disease [17], and pathologies of the kidneys [18,19]. Therefore, the ability of DKPs to reduce AGEs could potentially have beneficial effects on health, beyond those on skin aesthetics that could be of interest for cosmetic applications.

**Figure 1:**
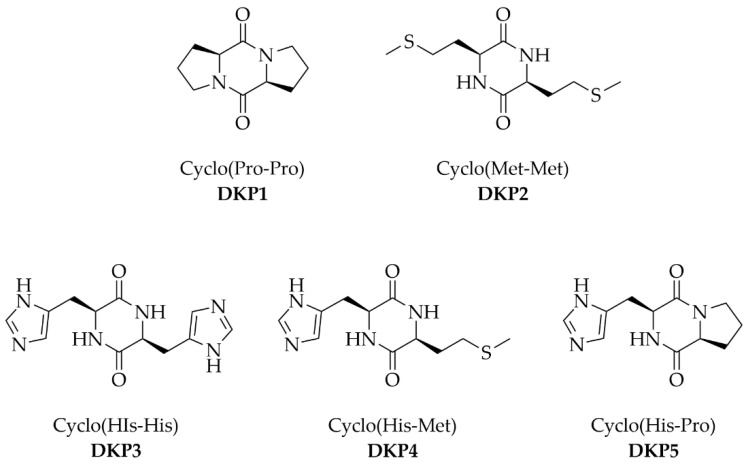
Five diketopiperazines (DKPs) used in this work.

In particular, 15 CDPs were recently tested in vitro for their anti-age properties, with the most active being cyclo(Leu-Leu), cyclo(Met-Met), and cyclo(Pro-Pro) [11]. In this work, we thus decided to test two of these as reference compounds, in addition with three more CDPs, i.e., cyclo(His-His), cyclo(His-Pro), and cyclo(His-Met), whose structures are shown in Figure 1. We reasoned that histidine ability to coordinate metals [20] could serve as a useful addition, for instance to reduce the occurrence of Fenton reactions [21] that occur during oxidative stress in ageing human skin [22]. The compounds shown below were thus synthesized using a green method in water in a microwave (MW) reactor, characterized by ^1^H-NMR, ^13^C-NMR, and ESI-MS spectroscopies and single-crystal X-ray diffraction, tested on fibroblasts for their cytocompatibility and anti-age properties, and assayed for their ability to penetrate the different layers on the skin in vitro.

## 2. Materials and Methods

### 2.1. Materials

(*N*-Boc)-l-prolyl-l-proline ((*N*-Boc)-Pro-Pro), l-methionyl-l-methionine (Met-Met), l-histidyl-l-histidine (His-His), l-methionyl-l-histidine (Met-His), and cyclo(His-Pro) (DKP5) were purchased from Bachem AG (Bubendorf, Switzerland), while all the other solvents and reagents were purchased from Merck (Milan, Italy). All materials were used as received without further purification. High-purity Milli-Q water (MQ water) was used to prepare all solutions and buffers, and it was obtained using a Milli-Q Academic System (Millipore RiOs/Origin purification system; St. Louis, MS, USA) with minimum resistivity 18.2 MΩcm^−1^. For the preparation of physiological solutions, sodium chloride, sodium hydrogenphosphate, and potassium dihydrogenphosphate were obtained from Carlo Erba (Milan, Italy). Mouse embryonic fibroblasts (NIH-3T3) were obtained from the Department of Life Sciences at the University of Trieste and were grown in complete Dulbecco’s Modified Eagle Medium (DMEM) supplied with 10% fetal bovine serum (100 U/mL penicillin, 100 mg/mL streptomycin (GIBCO^®^) and 2% antimycotic and antibiotic (GIBCO^®^). The 3-(4,5-dimethylthiazol-2-yl)-2,5-diphenyl- tetrazolium bromide (MTT) was purchased from Merck (Milan, Italy). The microwave (MW)-assisted synthesis was carried out in the Microwave reactor Discover SP–CEM Corporation. The sonicator used was the Branson Ultrasonics 3800 Series Ultrasonic Cleaning Bath. Franz cells were adapted for the skin absorption study. Porcine ears were purchased from the local butcher.

### 2.2. Synthesis of DKPs 1–4

Each linear dipeptide precursor was dispersed inside a MW glass vial at 30 mM with 1.0 mL of milliQ water, by ultrasonication in a water bath at 50 °C for a few minutes. The vial was placed in the MW reactor heated at 180 °C, 250 W, for 10 min. The reaction mixture was then freeze-dried to afford DKP1 (96% yield) and DKP4 (90% yield). In the case of DKP2 (70% yield) and DKP3 (80% yield), some racemization occurred and the homochiral DKPs were purified by RP-HPLC.

### 2.3. Purification of DKPs 2–3

DKP2 and DKP3 were purified by RP-HPLC, using a 1260 Agilent Infinity system, with semipreparative gradient pump (G1311B), semipreparative C-18 column (Kinetex, 5 m, 100 Å, 250 × 10 nm, Phenomenex, Torrance, CA, USA) and Photodiode Array detector (G1315C). DKP2 (R_t_ = 6.0 min) was dissolved in acetonitrile (MeCN)/water (30:70) + 0.05% trifluoroacetic acid (TFA) and purified with the following gradient (3 mL/min flow): t = 0–3 min, 25% MeCN; t = 16–18 min. A total of 95% MeCN. DKP3 (R_t_ = 5.5 min) was dissolved in water + 0.05% TFA and purified in isocratic conditions with 100% milliQ water (+ 0.05% TFA).

### 2.4. Single-Crystal X-ray Diffraction

Single crystals of DKP1 and DKP2 were collected with a loop, cryoprotected by dipping the crystals in glycerol, and stored frozen in liquid nitrogen. The crystals were mounted on the diffractometer at the Synchrotron Elettra (Trieste, Italy), beamline XRD1, using the robot available at the facility. The temperature was kept at 100 K by a stream of nitrogen on the crystals. Diffraction data were collected by rotating the crystal using the synchrotron radiation, wavelength 0.70 Å, rotation interval 0.5 °/image, crystal-to-detector distance of 85 mm. Further details can be found in the Supplementary Materials.

### 2.5. Live/Dead Cytotoxicity Assay

NIH-3T3 fibroblasts were seeded (10 k cells/well) on a μ-Slide Angiogenesis ibiTreat (Ibidi, Gräfelfing, Germany) in 30 μL medium (DMEM + 10% fetal serum albumin, 2% antimycotic and antibiotic from GIBCO) and cultured at 37 °C and 5% CO_2_ for 24 h, by handling the slides according to the manufacturer’s instructions. An amount of 1.5 μL of each DKP stock solution (2 mM in 10 mM sterile PBS, pH 7.4) was added to the wells to reach the desired final concentration of 0.1 mM. After 24 h, cells were treated with acridine orange (6 μL/well of a 20 μM solution in 50 mM PBS) and propidium iodide (3 μL/well of a 30 μM solution in 50 mM PBS). After 15 min, cells were imaged with a Leica microscope (DFC450C) with fluorescence green filter (exposure 200–400 nm, emission > 520 nm) with 10× and 40× objectives. Each condition was repeated in triplicate and each experiment was repeated twice (*n* = 6).

### 2.6. MTT Cytotoxicity Assay

NIH-3T3 fibroblasts were seeded (10 k cells/well) on 96-well microplates (Euroclone, tissue-culture grade treated, clear, flat bottom, sterile) in 100 μL of medium (DMEM + 10% fetal serum albumin, 2% antimycotic and antibiotic from GIBCO) and cultured at 37 °C and 5% CO_2_ for 24 h. Next, the medium was removed and exchanged with 100 μL of medium with serial dilutions of each DKP concentration (1.0 μM–1.0 mM) prepared in medium. A total of 1% SDS served as positive control (death). Cells were cultured for 24 h, then 10 μL of the MTT labelling reagent (Sigma, final concentration of 0.5 mL/mL) was added to each well, and the microplate was incubated for 4 h in a humidified chamber (37 °C and 5% CO_2_). Afterwards, 100 μL of the solubilization solution for formazan crystals (lysis buffer, 4 mM HCl + 0.1% IGEPAL in isopropanol) was added to each well, and the microplate was kept at room temperature while shaking (Rocker-shaker MR-12 Biosan, Vetrotecnica, Padova, Italy) for 30 min. The absorbance was read at 570 nm, with a reference wavelength at 690 nm (light scattering), using a multiwell plate reader (TECAN Infinite M1000 Pro). Two independent experiments were run in at least 3 wells (*n* = 6). Data are represented as mean ± standard deviation.

### 2.7. Skin Absorption Study

#### 2.7.1. Skin Membrane Preparation

Piglet ears were obtained from the local butcher and stored frozen at −25 °C for up to 4 months. Porcine skin was used as a model of human skin in the penetration test, because of its similarity in terms of morphology and permeability to human skin [23,24,25,26]. On the day of the experiment, they were thawed in physiological solution at room temperature and cut into 4 cm^2^ square pieces. The physiological solution was prepared by dissolving 2.38 mg Na_2_HPO_4_, 0.19 g KH_2_PO_4_, and 9 g NaCl in 1.0 L MilliQ water (final pH = 7.35). The thickness of pig-ear skin was measured with a micrometer caliper (Mitutoyo, Roissy en France, France) as <0.90 ± 0.02 mm. To evaluate skin integrity, trans epidermal water loss (TEWL) was measured on each skin piece after 1 h of equilibration using a Vapometer (Delfin Technologies, Kuopio, Sweden), already used previously [27]. The average TEWL values of skin samples was below 10 g m^−2^ h^−1^ [28].

#### 2.7.2. Donor Phase Preparation

Solutions of DKP1, DKP3, DKP4, and DKP5 were prepared by dissolving 3.0 mg of each DKP in 3.0 mL PBS to obtain a final concentration of 1.0 mg/mL. Before application in the donor chamber (DC), solutions were sonicated in an ultrasonic bath for a few minutes at room temperature. For DKP2, 5% EtOH was used in the PBS solution to assist with its dissolution, and a mild heating for a few minutes was used prior to deposition in the DC to remove the EtOH.

#### 2.7.3. In Vitro Permeation and Retention Study

Percutaneous absorption studies were performed in static diffusion cells according to OECD 2004 guidelines [29]. Skin membranes were mounted between the donor and receptor chambers of Franz-type diffusion cells with the stratum corneum (SC) facing the DC. The effective skin area was 0.95 cm^2^. The receptor fluid (RF) was composed of a freshly prepared PBS solution (0.1 M, pH 7.1) continuously stirred using a Teflon coated magnetic stirrer. The receptor compartment had a mean volume of 4.5 mL filled with RF. Mounted cells were maintained at 33 ± 1 °C by means of circulation of thermostated water in the jacket surrounding the cells [30]. At time 0, infinite dose of 0.5 mL DKP solution (1.0 mg/mL) was deposited in direct contact with the porcine skin surface in the Franz cell. This resulted in a theoretical applied dose of Q_0_ = 0.53 mg/cm^2^. The DC was sealed with parafilm during the whole duration of the experiment (6 h). The permeation study was carried out for 6 h to determine the permeation profile of DKPs remaining and permeating through the skin. At selected timepoints (0, 1, 2, 3, 4, 5, and 6 h), 0.15 mL of each RF were collected and analyzed. An equal volume of fresh RF was immediately replaced in each sample to maintain sink conditions. All experiments were conducted on 6 independent biological replicates. A skin sample with a PBS solution without DKP was used as a blank in each run. DKPs were quantified by LC-MS (see Section 2.7.5).

#### 2.7.4. Collection and Treatment of Samples

At the end of each experiment, Franz cells were dismantled and skin layers were separated. The non-absorbed fraction was removed from the skin surface by washing the DC thrice with 1.0 mL Milliq water for 20 s. Skin layers were separated as follows: the SC was isolated from viable layers by tape stripping (1 strip) using D-Squame tape (Monaderm, Monte-Carlo, Monaco), placed in vials each containing 5.0 mL MeOH and stirred overnight. Then, the explants (epidermis and dermis) were cut into small pieces with a scalpel and immersed in 2.0 mL MeOH and sonicated for 30 min. The samples were filtered (0.2 μm, nylon Uptidisc, 13 mm, Interchim, Montlucon, France) before LC-MS analysis. The total amount of DKPs in each extract was analyzed after 6 h by LC-MS. The same filtering procedure was applied to the RF samples obtained, as described in Section 2.7.3.

#### 2.7.5. DKP Quantification by LC-MS

The apparatus consisted of an Agilent 6120 Infinite system (Milan, Italy). Equipped with an analytical C-18 column (Luna, 5 μm, 100 Å, 150 × 2 mm, Phenomenex, Milan, Italy) at 35 °C a flow rate of 0.3 mL/min. Calibration curves were performed for each DKP and were linear from 0.1 to 5.0 mg/mL (R^2^ = 0.999998), with a LOD of 684.95 mUA s. For DKPs 1–3, the mobile phase consisted of MeCN and milliQ water with 0.5% formic acid and a gradient was used (t = 0, 25% MeCN; t = 30 min, 95% MeCN). For DKPs 4–5, the mobile phase consisted of ammonium acetate (2 mM) and MeOH with 0.5% formic acid and a gradient was used (t = 0, 100% or 90% ammonium acetate for DKP4 and 5, respectively; t = 12 min, 50% ammonium acetate).

#### 2.7.6. K_p_ Calculation and Statistical Analysis

K_p_ values were calculated by dividing the effective absorption rate by the equilibrium concentration of each DKP in the donor solution. The K**_p_** for each DKP was determined to compare the percutaneous kinetics. This parameter describes the membrane penetration.

Statistical analyses (*t* test) were performed in Excel and the level of significance (e.g., *p* < 0.05) is indicated in the legends of the figures that show the corresponding data and in the Supplementary Materials.

### 2.8. Anti-Age Assay

NIH-3T3 cells were seeded (7 k cells/well) in two sterile 96-well plates, flat-bottom, clear, in 100 μL medium (DMEM + 10% fetal serum albumin, 2% antimycotic and antibiotic from GIBCO) and cultured at 37 °C and 5% CO_2_ for 24 h. UV treatment was performed on one plate with a UV lamp (UVA 365 nm, 30 W/m^2^) for 55 min. The other plate was not irradiated and served as no-UV control. The medium was gently removed and replaced with an equal amount of fresh medium added with DKPs (5.1 μM) or without (blank). Cell culture was performed in a humidified chamber, as described above for another 24 h. The medium was then removed and cells gently rinsed with an equal volume of fresh PBS. Cells were fixed (40 μL of fixative solution containing PBS, 2% *v*/*v* formaldehyde, 0.2% *v*/*v* glutaraldehyde) for 3–5 min. Cells were rinsed twice with fresh PBS and stained with 50 μL/well of X-Gal solution (1 mg/mL X-Gal from Merck, 5 mM potassium ferricyanide, 5 mM potassium ferrocyanide, 2 mM MgCl_2_, 150 mM NaCl, citric acid/sodium phosphate buffer 40 mM, pH 6). Plates were incubated at 37 °C for 48 h and imaged under a brightfield microscope. Cells were counted and each condition was repeated in triplicate in two independent experiments (*n* = 6).

## 3. Results and Discussion

### 3.1. Synthesis and Characterization of DKPs 1–4

DKPs can be conveniently prepared in high yields using a green method based on the cyclization of the corresponding linear dipeptides in water using a microwave (MW) reactor [31]. Furthermore, it is also possible to use linear precursors that are protected at either or both termini, so that deprotection and cyclization occur in one-pot [32,33,34]. Indeed, when we applied this protocol to (*N*-Boc)-Pro-Pro, we obtained solely the desired cyclo(Pro-Pro) or DKP1 in 96% yield. In the case of DKPs 2–4, the unprotected linear precursors were also successfully converted in the desired DKPs in high yields (70–90%). Each DKP purity and identity was confirmed by ^1^H-NMR, ^13^C-NMR, and ESI-MS analysis (see Supplementary Materials).

### 3.2. Single-Crystal X-ray Diffraction Analysis

Several crystallization trials were performed for all DKPs. However, single crystals of suitable quality for X-ray diffraction were obtained solely for DKP1 and DKP2. DKP1 crystallized at 10 mM and 20 mM in dodecane, while DKP2 crystallized in the solvent mixture was used for HPLC purification (see Section 2.3).

In terms of the DKP-ring conformation, both DKPs adopt a boat conformation, which is the typical case for 3,6-disubstituted, C_2_-symmetric DKPs, although sometimes the boat can be slightly flattened [10]. The ring of DKP2 adopts a boat conformation, both in the low-temperature and room-temperature crystal structures [35], although there is not a perfect symmetry. Analogous is the case of DKP1, with the β carbons at quasiequatorial positions, in agreement with previous studies [36,37]. This conformer has been calculated to be the most stable, and lower in energy by 1.3–1.7 kcal/mol relative to the planar one [38,39]. However, in solution at room temperature, the boat and the planar conformers can interconvert into each other [10].

**Figure 2:**
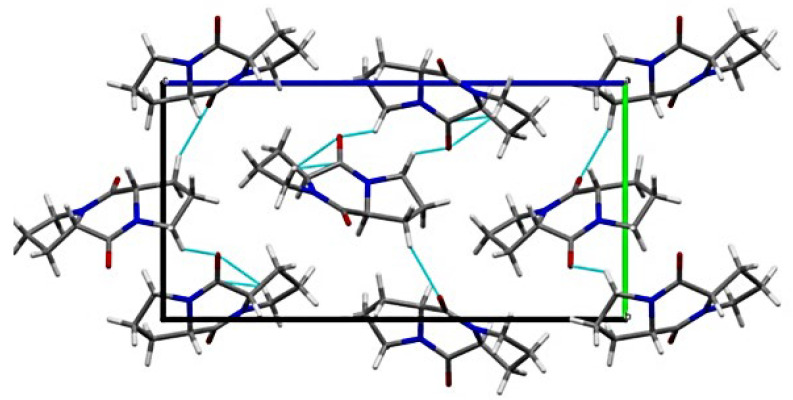
Crystal packing of DKP1 (CCDC 2203140).

The packing of DKP1 is shown in Figure 2. Despite the presence of only one molecule in the asymmetric unit, the solid-state assembly appears to be more complicated than the one of DKP2 (vide infra). Indeed, the crystal packing analysis reveals the presence of two different H-bonding patterns. Both are based on CO∙∙∙HC interactions, with the former involving mainly the O1 oxygen atom, which interacts with two different DKP molecules, while the latter involves the O2 oxygen atom.

**Figure 3:**
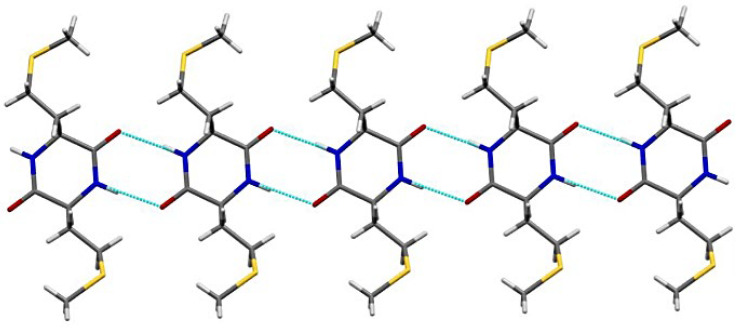
H-bonded ribbons of DKP2 (CCDC 2203142).

In the case of DKP2, the structure revealed a polymorph relative to the one originally reported by Tamburro and coworkers [40], and later by Mendham et al. [35]. Notably, this new crystal structure features a different space group (P2_1_) and two chemically equivalent, but crystallographically independent, molecules in the unit cell. In the previously reported crystal structures, the DKP ring adopts a twisted boat conformation with a fold angle of 30°. In the present polymorph, the DKP ring is almost planar with a fold angle of −78°. The crystal packing is characterized by NH∙OC H-bonded ribbons (Figure 3).

### 3.3. Cytocompatibility Assays In Vitro

**Figure 4:**
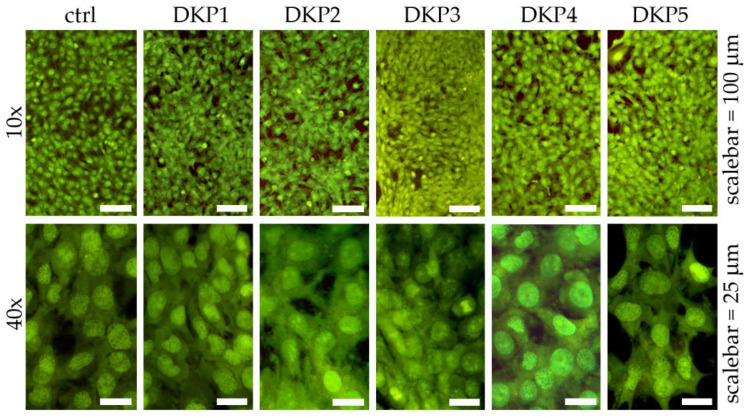
Live/dead assay on fibroblast cells cultured with the five DKPs for 24 h.

DKPs occur naturally, and their biosyntheses and biological activities have been widely studied in light of their promising potential for biomedical applications [41]. In this work, their cytocompatibility was tested in vitro on fibroblast cell cultures, using live/dead and metabolic (i.e., MTT) assays. Cell cultures of fibroblasts were exposed for 24 h to each DKP, and live/dead assays revealed very high viability in all cases that were not significantly different relative to the control (Figure 4).

**Figure 5:**
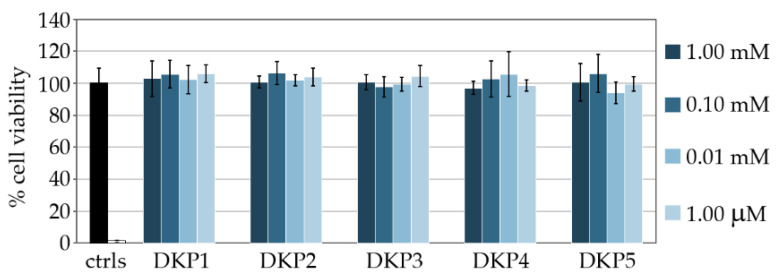
MTT assay on fibroblast cells cultured with the five DKPs at different concentrations.

Metabolic assays were also performed to confirm the good cytocompatibility of DKPs, using 3-(4,5-dimethylthiazol-2-yl)-2,5-diphenyltetrazolium bromide (MTT). A total of 1% SDS served as positive control (i.e., dead cells, white column in Figure 5). All DKPs were assayed in a range of concentrations spanning from 1.0 μM to 1.0 mM and no significant difference relative to controls was noted (Figure 5).

### 3.4. Skin Absorption Studies of DKPs

The human skin is composed of three layers, which are the epidermis, the dermis, and the hypodermis [42]. The epidermis is avascular, stratified, squamous epithelium, and its outer layer is the stratum corneum (SC) [43]. The dermis is mainly composed of fibroblasts in an extracellular matrix of proteins [44]. Finally, the hypodermis is composed mainly of subcutaneous fat with blood and lymphatic vessels [45]. Ideally, cosmetics should surpass the barrier composed of the SC, and reach the dermis for maximal efficacy, without passing over and potentially leading to systemic effects [46].

The majority of substances penetrate the skin through passive diffusion, which can be described most simply by Fick’s diffusion law [47]. As a general rule, molecules that more readily permeate the skin have a molecular weight (MW) under 5 kDa and an octanol-water partition coefficient logP = 1–3 [48]. The logP of the five DKPs is shown in Table 1.

In this work, we used ear pigskin as an ex-vivo model to study skin absorption [24,26] in Franz diffusion cells for 6 h, after which the amount of DKPs was quantified by LC-MS in the donor compartment (DC), in the receptor fluid (RF), and in the various skin layers (Table 2).

Skin absorption is a process that describes the passage of compounds across the skin and includes penetration and permeation. Dermal penetration is the mass of the test substance that enters the skin, and dermal permeation is the mass that has transferred from the skin to the reservoir compartment fluid [49]. Instead, permeability is the velocity of passage of a drug through a biological lipid membrane [50]. The data analysis of the present study revealed that the effective dose of DKP in each donor compartment (DC), expressed in mg/cm^2^, were respectively 0.31 ± 0.07 mg/cm^2^ for DKP1; 0.38 ± 0.03 mg/cm^2^ for DKP2; 0.11 ± 0.01 mg/cm^2^ for DKP3; 0.20 ± 0.02 mg/cm^2^ for DKP4 and 0.38 ± 0.04 mg/cm^2^ for DKP5 (Table 2). These results were further used for the permeability coefficient (K_p_) calculation. In all cases, the DKP amounts found in the RF were quite similar, ranging from ~ 10% for DKP3 and DKP5, to 23–30% for DKP1, DKP2, and DKP4, relative to the applied dose of 0.53 mg/cm^2^. As expected, no DKP was detectable in the blank (not shown).

In addition, the distribution of each DKP in the various skin layers was also assessed post-exposure. DKP5 was the only DKP found in the SC (0.09 ± 0.00 mg/cm^2^), which had also the highest amount relative to the other skin layers (i.e., epidermis and dermis). Conversely, all the other DKPs were found exclusively in the epidermis and dermis layers, with the highest penetration being measured for DKP2, followed by DKP1, and corresponding to the total recovered in the skin (Table 2). These were also the two most hydrophobic DKPs of the series, according to their logP values (Table 1). Conversely, the more hydrophilic DKP3 and DKP4 displayed the lowest skin penetration, corresponding to 0.05 ± 0.001 mg/cm^2^. It is also well known that a hydrophilic substance cannot penetrate the skin easily because it cannot enter the hydrophobic SC layer, while a hydrophobic substance easily enters the SC, but it remains stored inside it since the next layer is hydrophilic [51]. Statistically significant differences (*p* < 0.05) were found between DKP samples (see Supplementary Materials).

Furthermore, considering the effective dose, the permeability coefficient K_p_ (cm/h) of each DKP was determined by dividing the effective absorption rate by the equilibrium concentration of each DKP in the donor solution. Apparently, the highest K_p_ was observed for DKP1 ranging around 4.59 ∙ 10^−1^ cm/h, while DKP5 was the least permeable DKP with K_p_ measured in the range of 0.85 ∙ 10^−1^ cm/h. This parameter allows us to compare the percutaneous kinetics, which revealed significantly different absorption rates in the increasing order DKP5 < DKP4 < DKP2 < DKP3 < DKP1, showing a general trend, whereby the most hydrophilic DKPs (DKP4-DKP5) had the lowest K_p_, and vice versa. The only exception to the trend was DKP3, and this fact can be explained with the observation that DKP3 is the only one with two aromatic sidechains that are likely to coordinate metal cations (e.g., Zn^2+^ and Cu^2+^) that are naturally present in biological samples, thus affecting its hydrophilicity [52,53].

In conclusion, all the data point to DKP5 being the one that is most slowly absorbed, and it is also the only one found in the SC at the end of the experiment. The other DKPs 1–4 all displayed higher permeation rates and the ability to surpass the SC and reach the deeper layers of the skin, i.e., the epidermis and dermis. Clearly, formulation design will be key in the future to modulate their penetration rate in the skin, as needed for the intended application.

### 3.5. Anti-Age Activity

Andrè et al. recently reported the ability of DKPs 1–2 to reduce AGEs in fibroblasts by 27 ± 1% and 28 ± 1%, respectively, relative to the controls [11]. We couldn’t find any further data related to DKP anti-age activity in the literature, and thus we used these two compounds as reference in our experiments.

**Figure 6:**
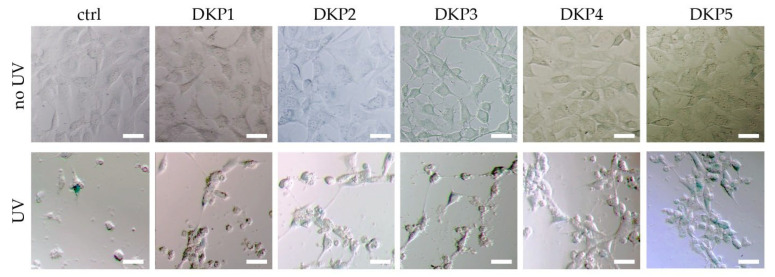
Anti-age assay without (ctrl) or with DKPs 1–5 on fibroblast cells, without (**top panels**) or with (**bottom panels**) UV exposure. Scalebars = 20 microns.

The ageing assay was based on UV exposure of cultured fibroblast cells, and subsequent treatment with the five DKPs (see Section 2.8 for details). A visual inspection by optical microscopy revealed that without UV treatment, no significant differences were noted between samples treated with or without any DKP (Figure 6, top row), thus, confirming the good cytocompatibility observed by the live/dead and MTT assays. UV treatment significantly reduced cell viability in all cases, with the most negative effects observed for the control (Figure 6, bottom row).

**Figure 7:**
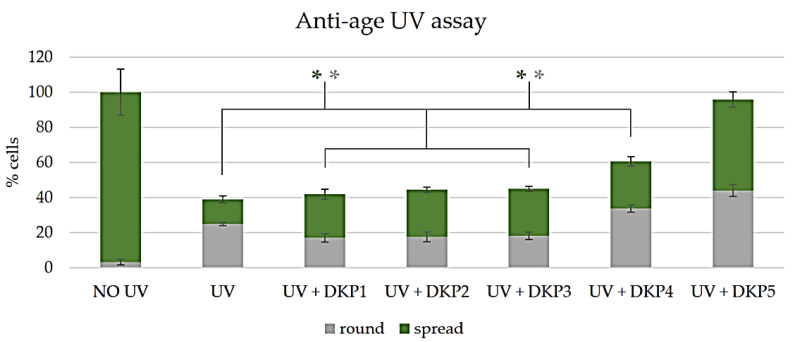
Quantitative anti-age assay with/without DKPs 1–5 on fibroblast cells. The asterisk denotes *p* < 0.05 between spread cells (green *) or round cells (gray *).

The reported anti-age activity of DKP1 and DKP2 was confirmed, as the numbers of viable, spread cells (green bars in Figure 7) nearly doubled relative to the UV-treated control without DKPs. We were very pleased to see that the three new DKPs performed as well (DKP3), or significantly better (DKP4-DKP5). A significant increase in total cell numbers was noted only for treatment with DKP4 and DKP5, with the latter leading to a further doubling of spread cells (Figure 7).

**Figure 8:**
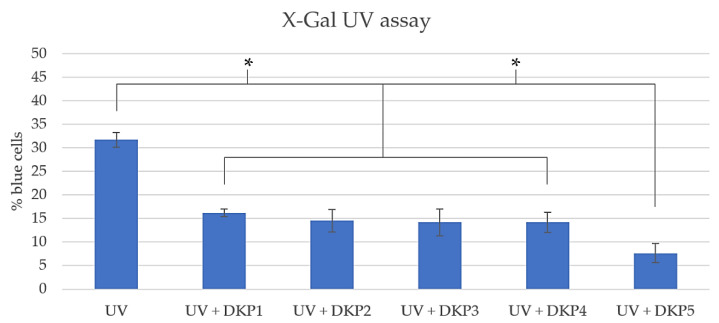
Quantitative X-Gal assay on UV-treated cells without or with DKPs 1–5. * denotes *p* < 0.01.

The X-Gal assay was performed too, since this substrate is converted into a blue product by β-galactosidase, which is a senescence biomarker [54]. This convenient assay revealed a similar extent of protective activity from UV-induced cell senescence for DKPs 1–4 (*p* > 0.01) and higher anti-age activity for DKP5 (Figure 8). Overall, this assay confirmed the results described above, which all point to DKP5 as the best DKP of the series in terms of anti-age activity.

Interestingly, DKP5 is known to be endogenous to blood, cerebrospinal fluid, brain, spinal cord, semen, and gastrointestinal tract of humans [55]. Part of it derives from the enzymatic degradation of the thyrotropin-releasing hormone, while the origin of the rest remains to be elucidated [56]. Interestingly, its administration reduces food intake [57]. This DKP also has other pharmacological activities, such as regulation of body temperature, inhibition of prolactin secretion, and modulation of motor functions, which may be exerted by affecting central amine transport mechanisms [58]. Indeed, this DKP has the remarkable ability to pass the blood–brain barrier by passive diffusion [59], and it has been widely studied for its ability to exert a protective role in neurodegenerative and metabolic diseases [60]. Given its high potential for therapeutic use by oral administration also, it has been proposed as an important innovative tool to counteract neuroinflammation-based degenerative diseases [61].

The exact mechanism of action is unclear, but administration of DKP5 with zinc stimulated the synthesis of insulin degrading enzyme [62], overall leading to better control of glycaemia and Aβ levels, and, more generally, affecting proteostasis [63]. The non-enzymic mechanisms of its antioxidant properties include radical scavenging and metal chelation to reduce oxidative reactions, and carbonyl quenching to detoxify carbonyl species, an activity that has been indirectly ascribed to DKP5, as it requires the free amino group that originates from the DKP-ring opening [64].

## 4. Conclusions

This work demonstrated that a convenient green cyclization in water of linear dipeptides can be applied with high yields to produce cyclo(Pro-Pro), cyclo(Met-Met), cyclo(His-His), and cyclo(His-Met). Spectroscopic and X-ray diffraction analyses confirmed the identity and purity of all DKPs studied. Their cytocompatibility in fibroblast cell culture was excellent for all tested concentrations up to 1.0 mM. Furthermore, the anti-ageing activity of DKP1 and DKP2 was confirmed, and it was reported for the first time for the other three DKPs, with results as good (for DKP3) or even better (for DKP4-DKP5), especially for DKP5, which is an endogenous DKP with excellent therapeutic potential and the added benefit of being able to pass the blood–brain barrier to also exert effects in the central nervous system.

Finally, the ability of the five DKPs to penetrate the skin was evaluated in vitro using Franz cells on piglet ear model samples. It was demonstrated that all DKPs can penetrate the skin with significantly different permeability rates, the lowest one being for DKP5. Clearly, the skin absorption ability of this DKP will need to be enhanced through advanced formulations, to be able to fully exploit its anti-ageing activity for cosmetic use.

In conclusion, this study adds three more DKPs to the pool of anti-ageing cyclodipeptides with excellent cytocompatibility and the ability to penetrate the skin. Future studies may be directed towards the development of suitable formulations to maximize their ability to be enriched in the dermis for cosmetic use, or elsewhere for systemic effects, given the established health benefits from DKP5 administration. For instance, combination with linear short peptides that can self-assemble into nanostructured hydrogels and mimic natural components of the extracellular matrix [65] could offer a promising avenue for cosmetic formulations. The mechanistic elucidation of the observed effects in terms of cell protection from UV damage certainly deserves further investigation to better understand how these biomolecules exert their bioactivity, so that their use can be extended beyond cosmetics, to the preservation of human health.

## Figures and Tables

**Table 1:** Physico-chemical properties of the five DKPs.

DKP	Cyclodipeptide	logP ^1^	MW (Da)	Ionizable Groups ^2^
DKP1	Cyclo (Pro-Pro)	−0.89	194	0
DKP2	Cyclo (Met-Met)	−0.64	262	0
DKP3	Cyclo (His-His)	−3.60	274	2
DKP4	Cyclo (His-Met)	−2.12	268	1
DKP5	Cyclo (His-Pro)	−2.24	234	1

^1^ Calculated with ChemDraw Professional v.15.0.0.106, Perkin-Elmer. ^2^ Number of ionizable groups in the DKP sidechains that can lead to charged species, depending on the pH value.

**Table 2:** Quantification of DKPs in the skin absorption experiment. DC = donor compartment. E + D = epidermis + dermis. SC = stratum corneum. RF = receptor fluid. K_p_ = permeation coefficient.

DKP	DC (mg/cm^2^)	Skin (E + D, mg/cm^2^)	Skin (SC, mg/cm^2^)	Total Skin (mg/cm^2^)	RF (mg/cm^2^)	K_p_ (cm/h)
DKP1	0.31 ± 0.07	0.010 ± 0.003	0.000 ± 0.000	0.010 ± 0.003	0.16 ± 0.05	4.59 10^−1^
DKP2	0.38 ± 0.03	0.020 ± 0.007	0.000 ± 0.000	0.020 ± 0.007	0.13 ± 0.02	3.61 10^−1^
DKP3	0.11 ± 0.01	0.005 ± 0.001	0.000 ± 0.000	0.005 ± 0.001	0.04 ± 0.02	4.00 10^−1^
DKP4	0.20 ± 0.02	0.004 ± 0.000	0.000 ± 0.000	0.004 ± 0.000	0.12 ± 0.03	1.82 10^−1^
DKP5	0.38 ± 0.04	0.005 ± 0.001	0.009 ± 0.000	0.014 ± 0.001	0.07 ± 0.03	0.85 10^−1^

## Data Availability

Data are provided in the Supplementary Information and further information is available from the authors upon request.

## Supplementary Materials

The following supporting information can be downloaded at: www.mdpi.com/article/10.3390/biomedicines10102342/s1, including spectroscopic characterization and further data.
