# Supplementary material for: Cyclodipeptides: From Their Green Synthesis to Anti-Age Activity
Source: Biomedicines. 2022 Sep 20;10(10):2342. doi: 10.3390/biomedicines10102342 (PMC9598056; doi:10.3390/biomedicines10102342)
Supplement: Supplementary file 1 [file biomedicines-10-02342-s001.zip › biomedicines-1908347-supplementary.pdf]

biomedicines

MDPI

Supplementary Materials Cyclodipeptides: From Their Green Synthesis to Anti-Age Activity

Veronica Mosetti, Beatrice Rosetti, Giovanni Pierri, Ottavia Bellotto, Simone Adorinni, Antonella Bandiera, Gianpiero Adami , Consiglia Tedesco, Matteo Crosera, Greta Camilla Magnano and Silvia Marchesan

1. Cyclo(Pro-Pro) (DKP1) spectroscopic data

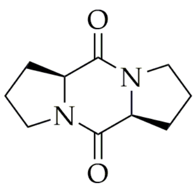

Cyclo(Pro-Pro)
DKP1

[1]**H NMR** (400 MHz, CD_3OD, TMS), δ (ppm): 4.34 (dd, *J* = 8.5 Hz, *J* = 7.5 Hz, 2H, αCH), 3.55 – 3.43 (m, 4H, δCH_2), 2.33 – 2.25 (m, 2H, βCH), 2.12 – 1.88 (m, 6H, βCH and γCH_2). [13]**C NMR** (100 MHz, CD_3OD, TMS), δ (ppm): 168.6 (2 x CO); 61.7 (2 x αC); 46.2 (2 x δC); 28.7 (2 x βC); 24.2 (2 x γC). MS (ESI): m/z 195.1 $(M+H)^+$.

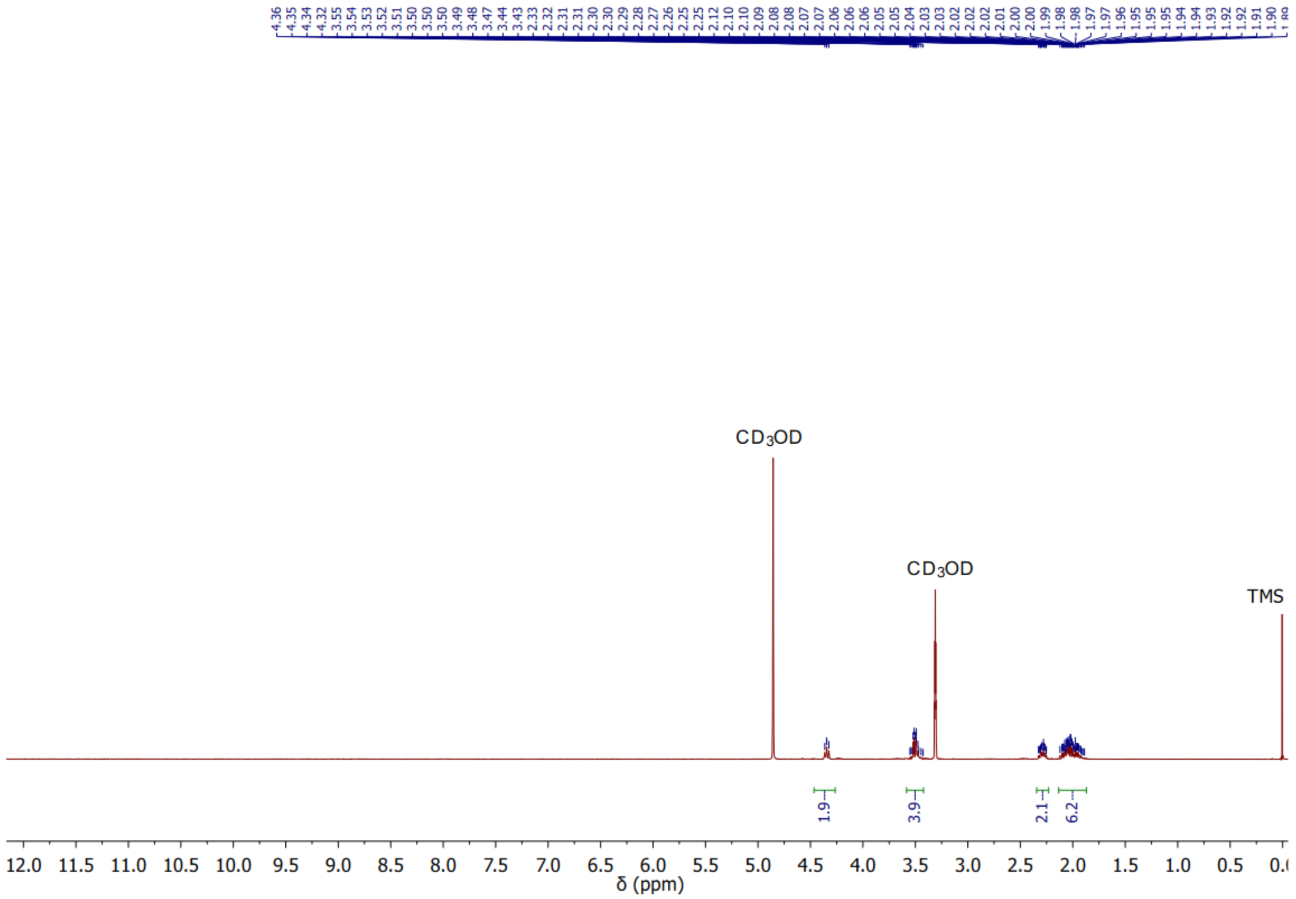

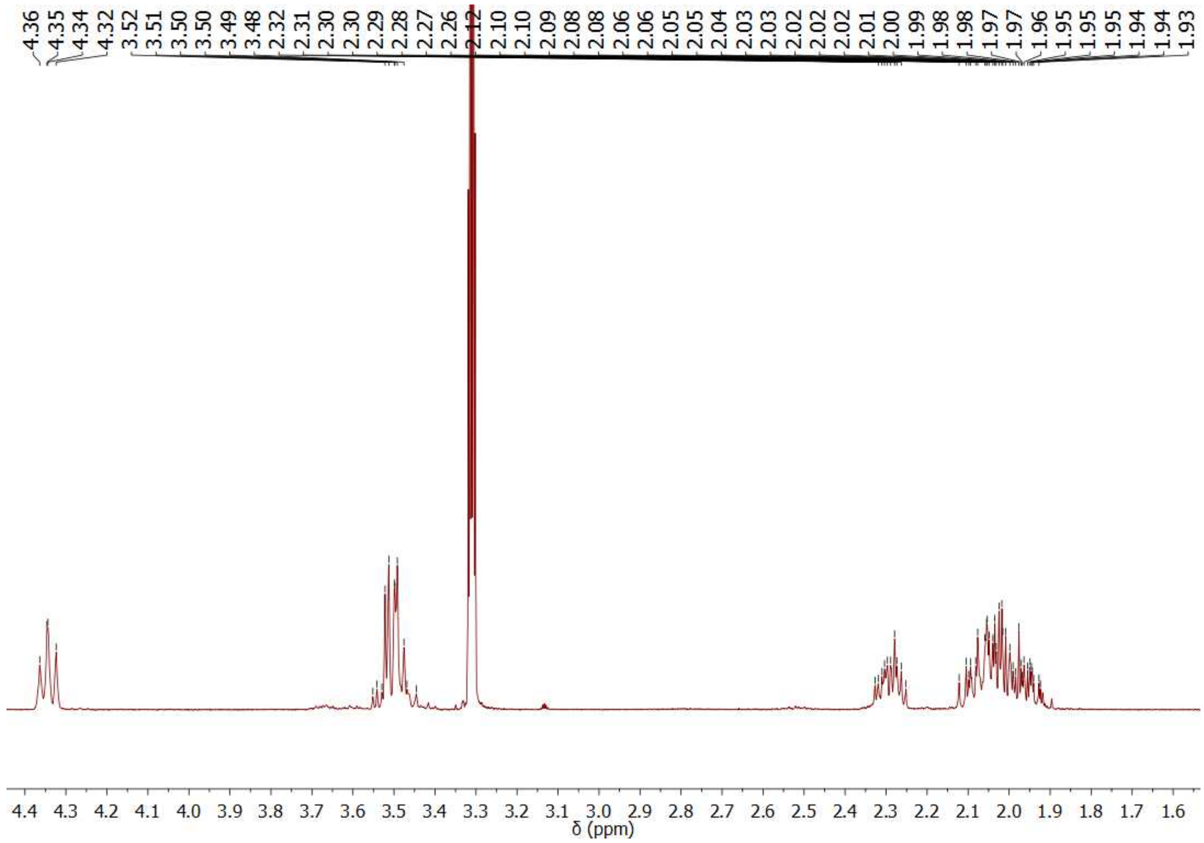

Figure S1. ^{1}H-NMR spectrum of DKP1 (full-view, top; enlarged view, bottom).

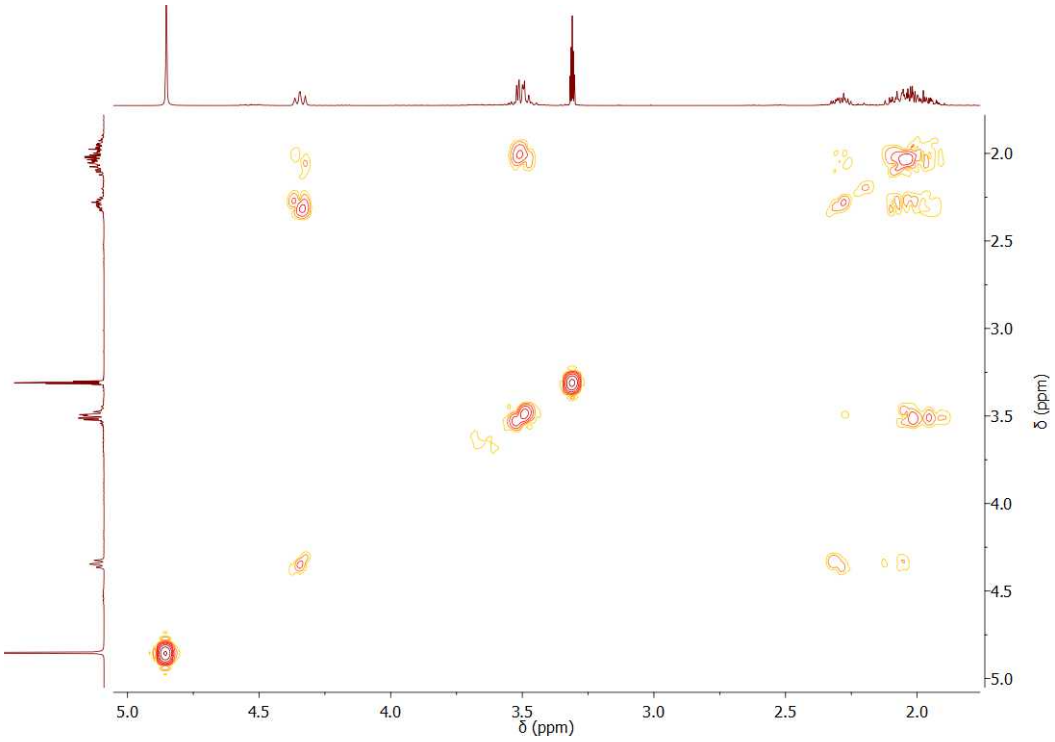

Figure S2. gCOSY 2D-NMR spectrum of DKP1.

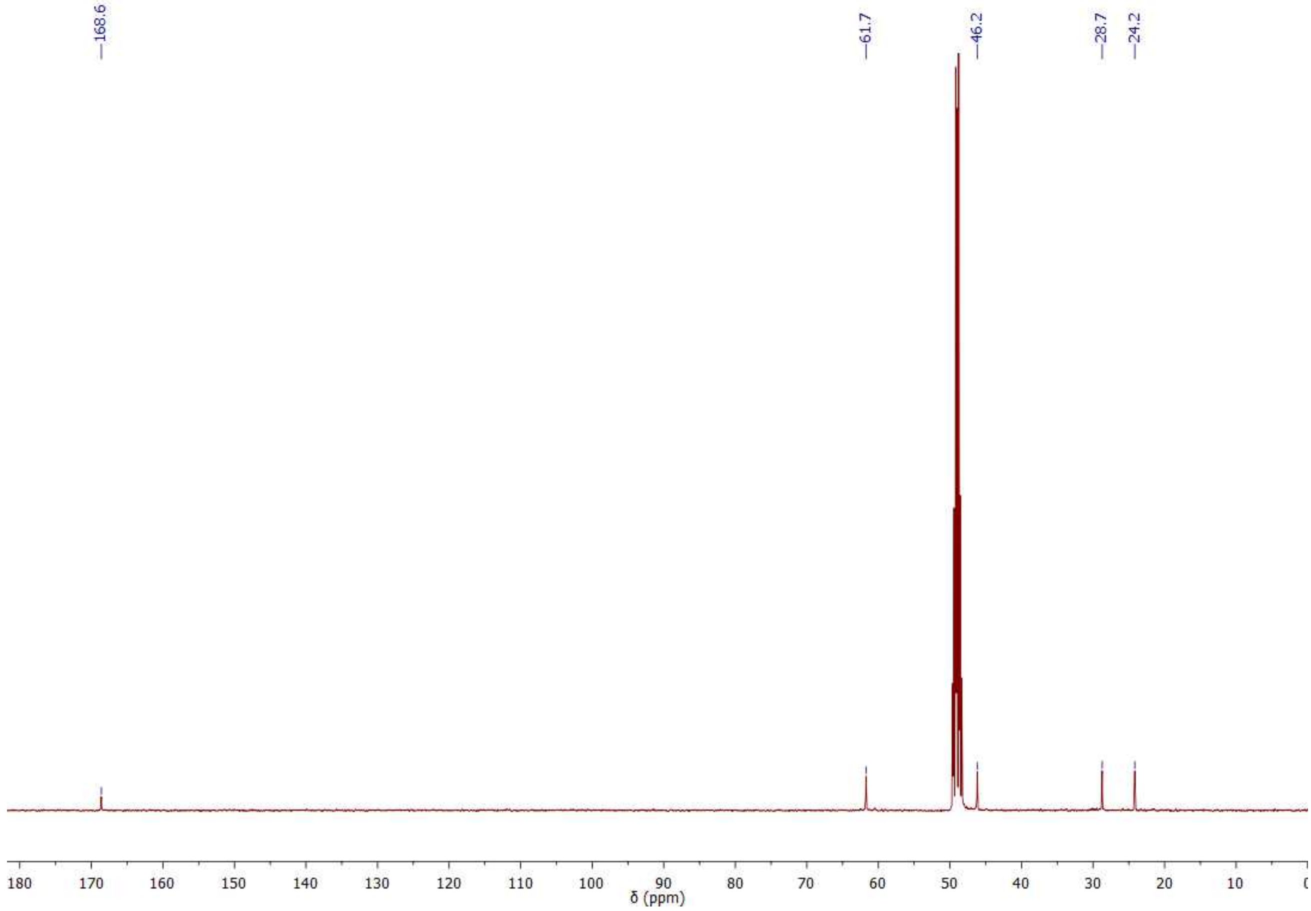

Figure S3. ^{13}C-NMR spectrum of DKP1.

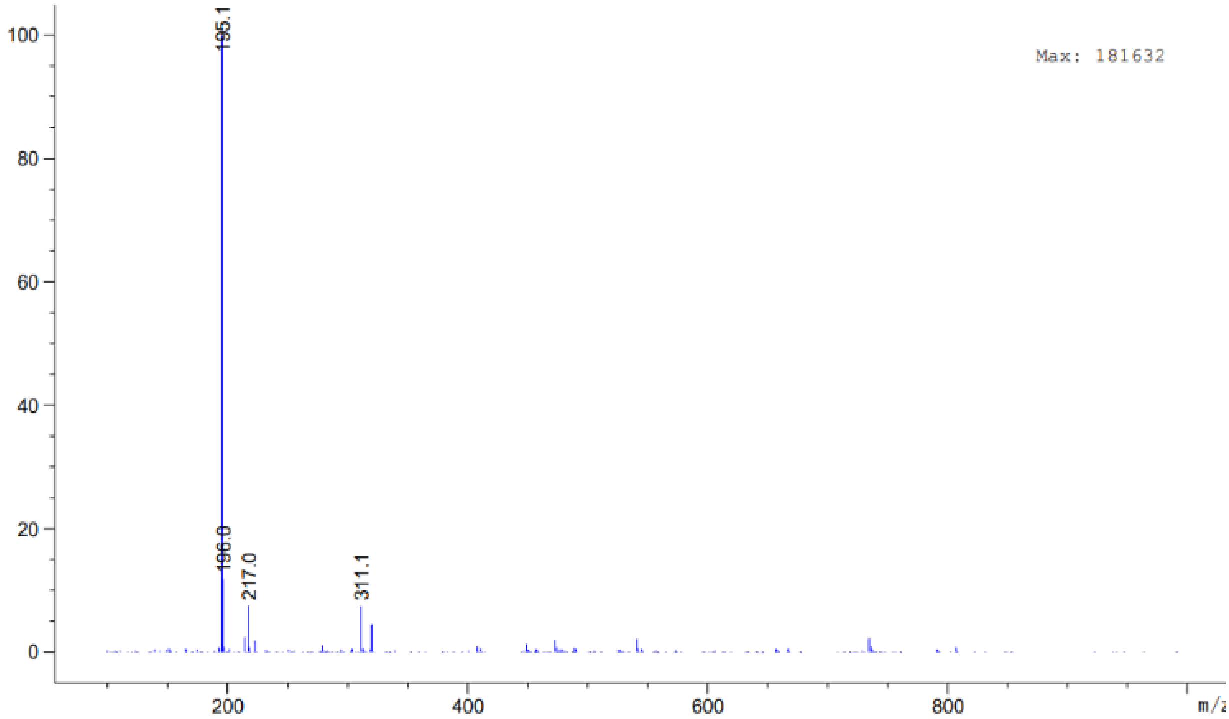

Figure S4. ESI-MS spectrum of DKP1 (positive ion mode).

2. Cyclo(Met-Met) (DKP2) spectroscopic data

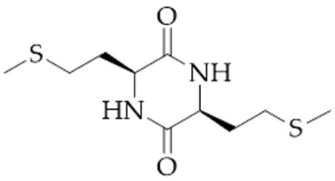

Cyclo(Met-Met)
DKP2

^{1}**H NMR** (400 MHz, CD_3OD, TMS), δ (ppm): 4.13 (dd, *J* = 8.0 Hz, 2H, αCH), 2.61 (dd, *J* = 7.4 Hz, 4H, γCH_2), 2.18 – 2.02 (m, 4H, βCH_2), 2.10 (s, 6H, 2 x CH_3). ^{13}**C NMR** (100 MHz, CD_3OD, TMS), δ (ppm): 175.3 (2 x CO); 60.0 (2 x αC); 39.6 (2 x βC); 35.3 (2 x γC); 20.1 (2 x δC). MS (ESI): m/z 263.0 $(M+H)^+$, 285 $(M+Na)^+$, 215 $(M-SCH_3)^+$, 167 $(M-(SCH_3)_2+H)^+$.

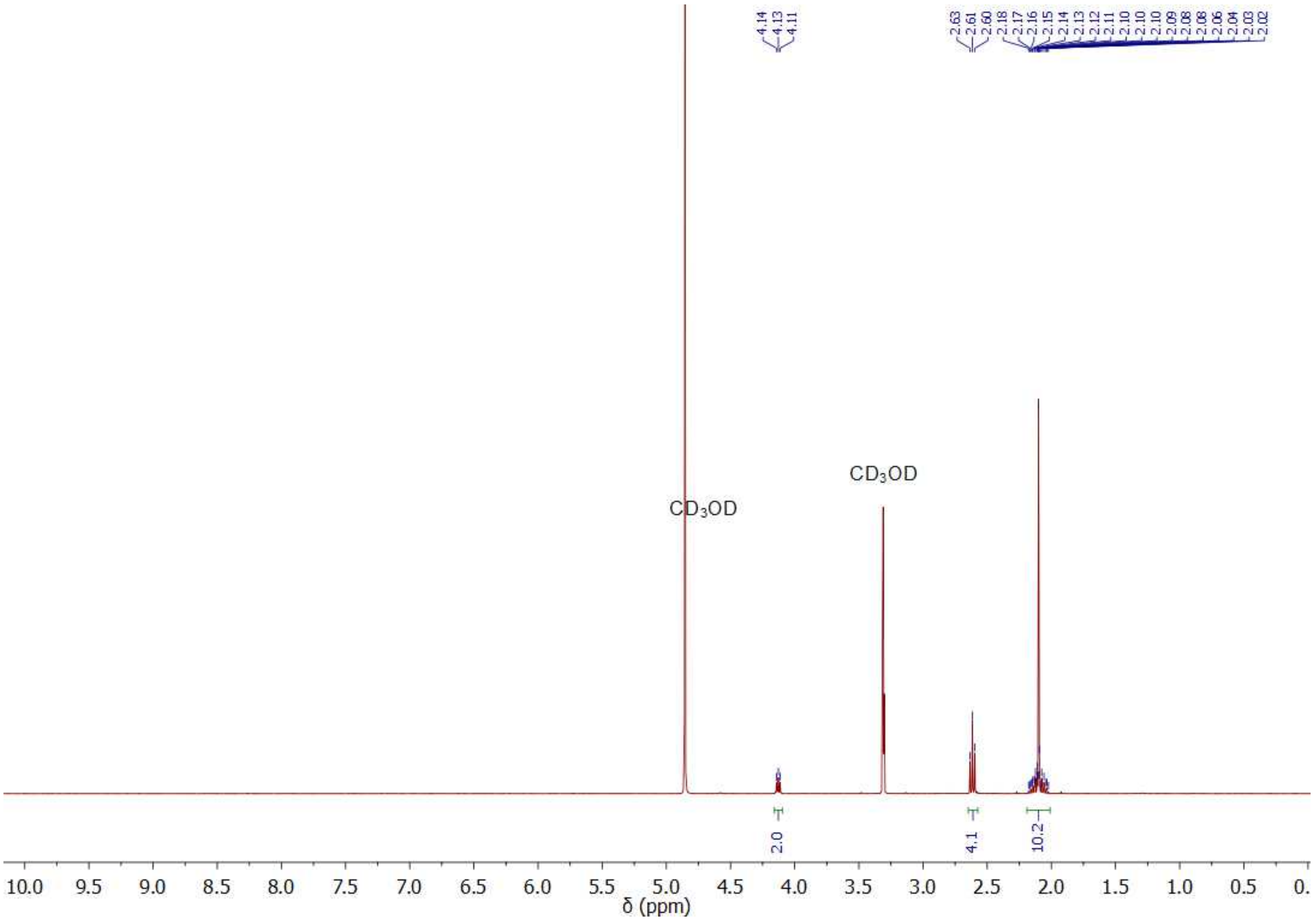

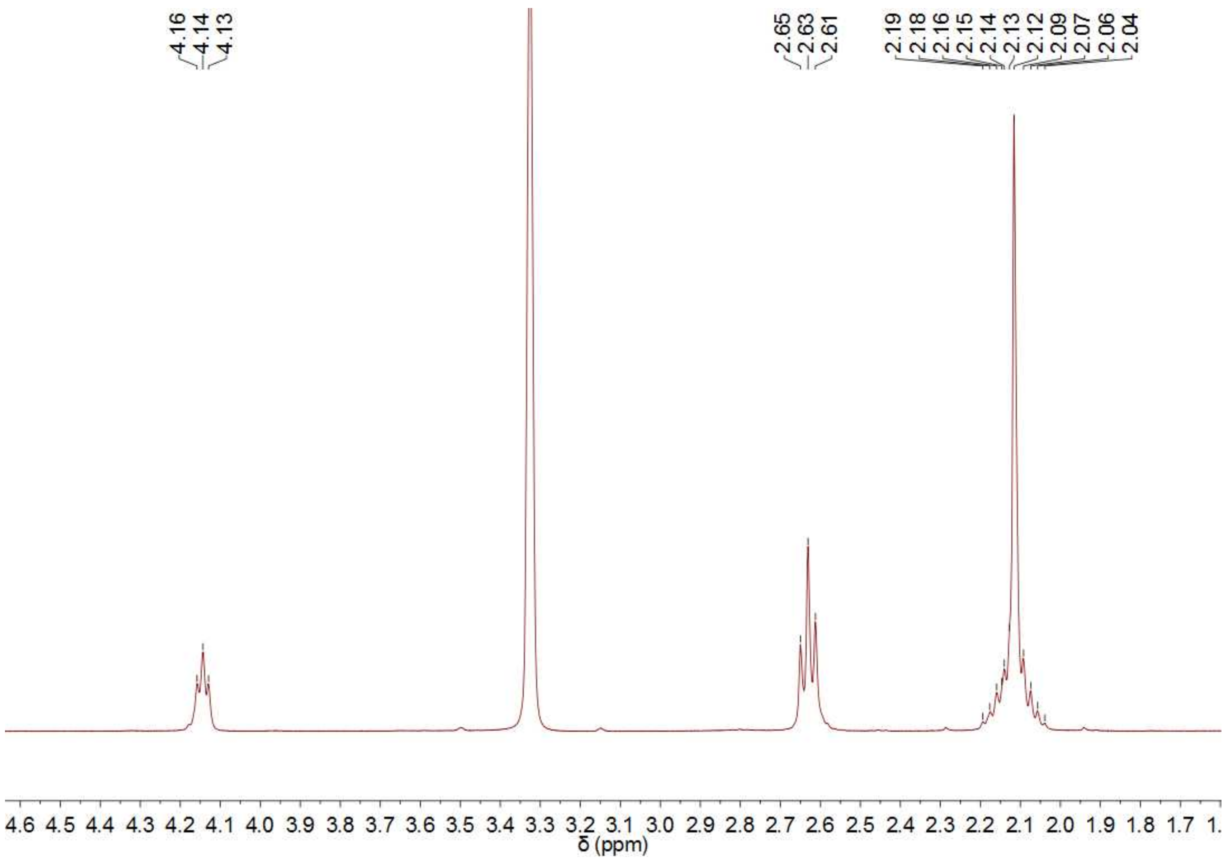

Figure S5. ^{1}H-NMR spectrum of DKP2 (full-view, top; detailed-view, bottom).

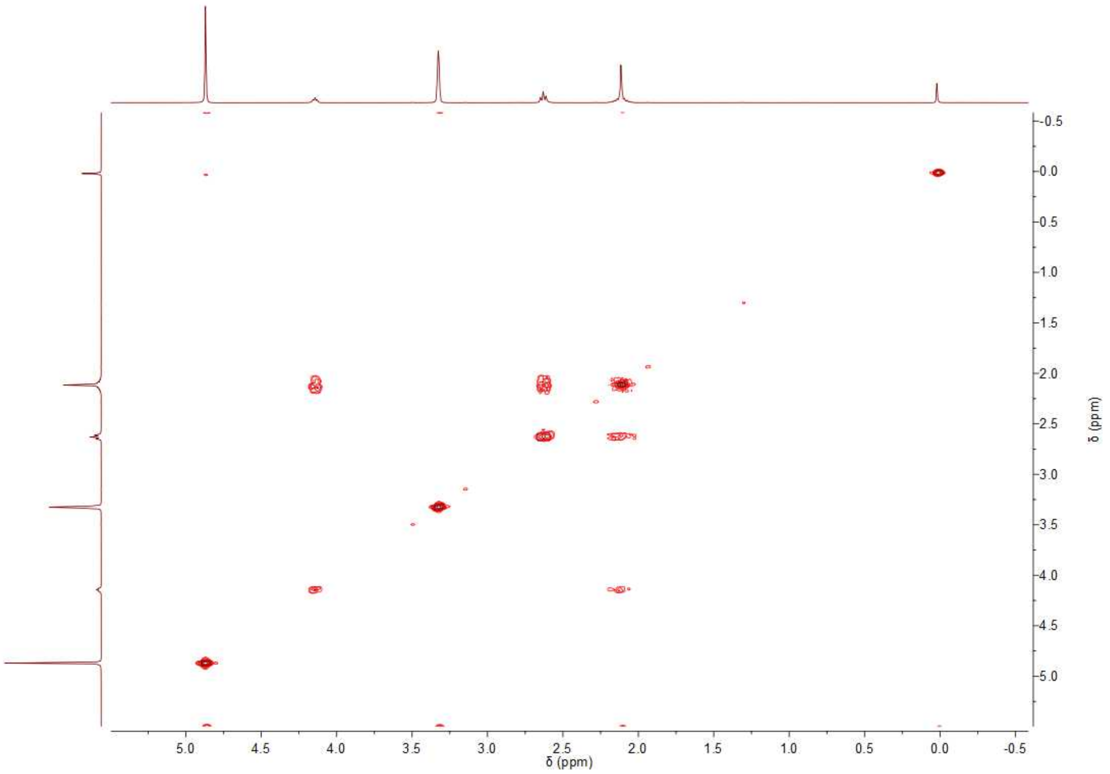

Figure S6. gCOSY 2D-NMR spectrum of DKP2.

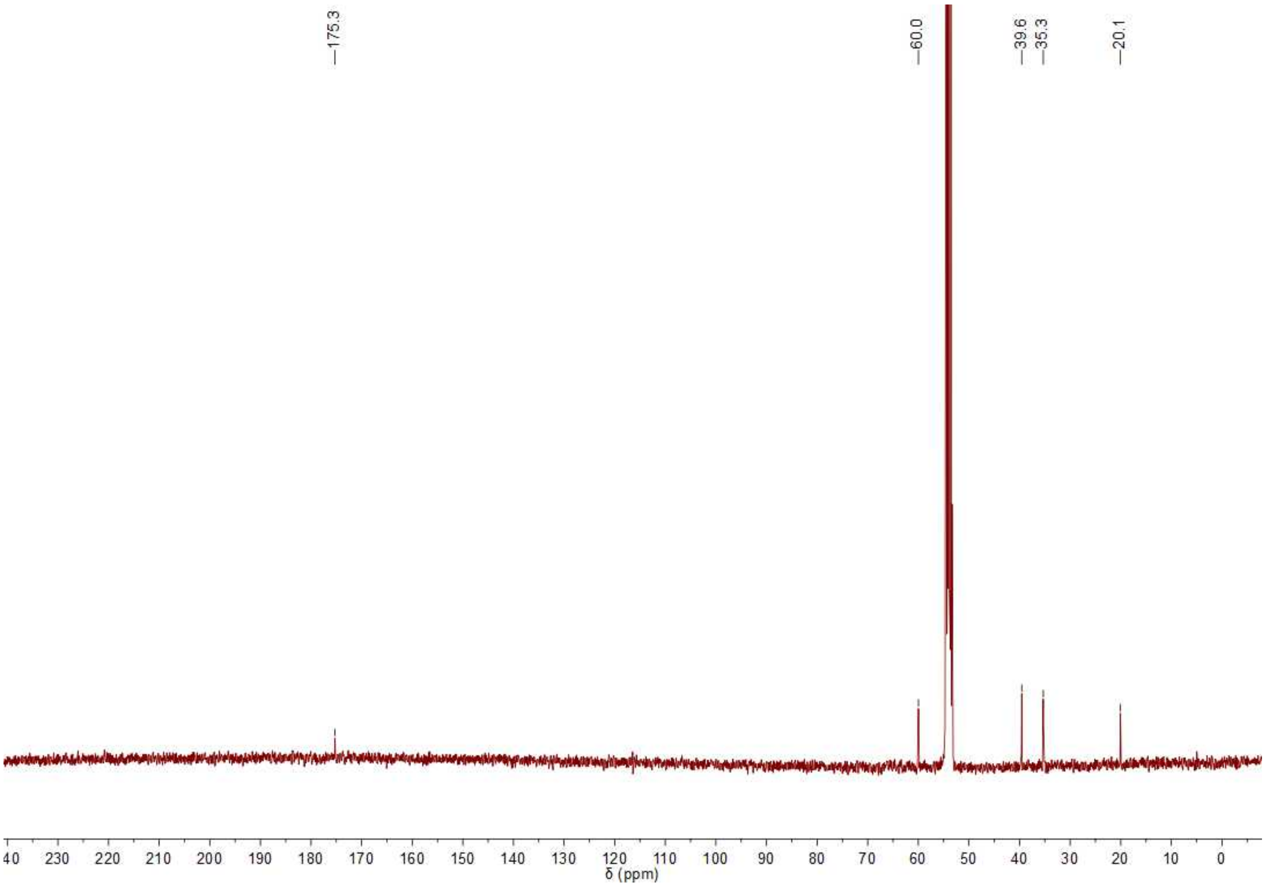

Figure S7. ^{13}C-NMR spectrum of DKP2.

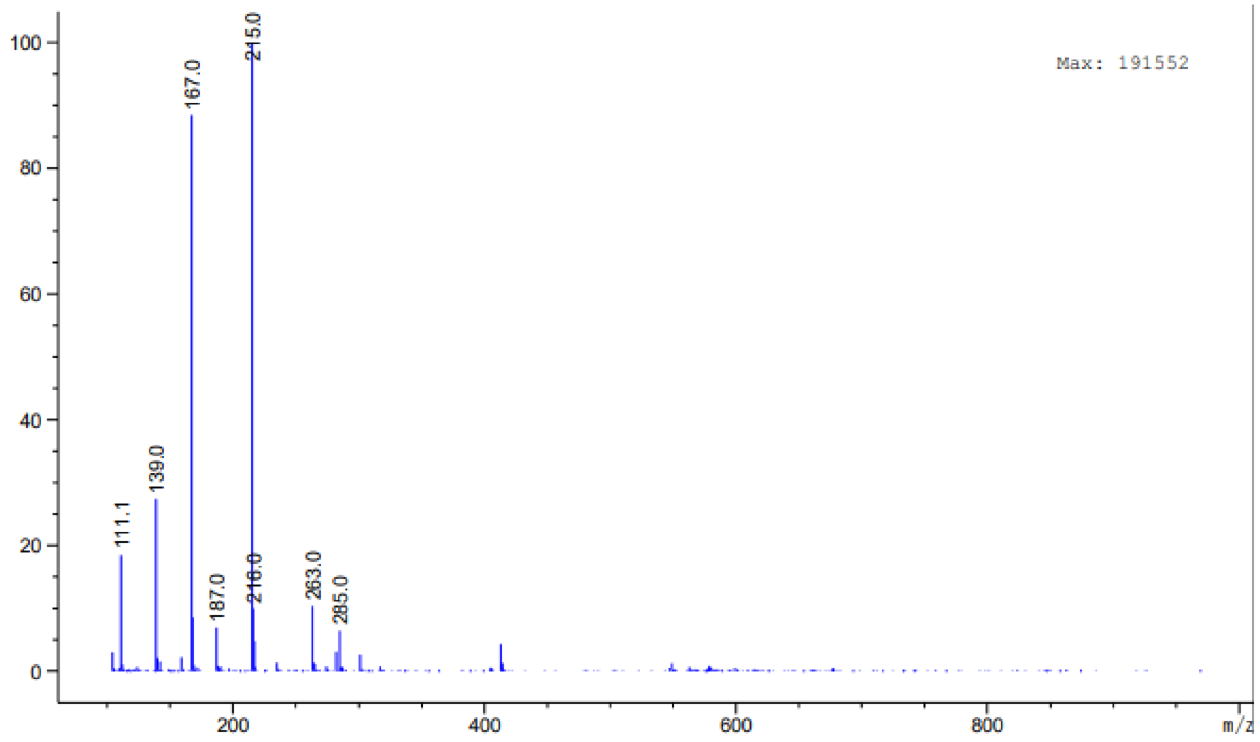

Figure S8. ESI-MS spectrum of DKP2 (positive ion mode).

3. Cyclo(His-His) (DKP3) spectroscopic data

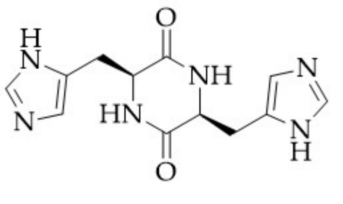

Cyclo(His-His)
DKP3

^{1}H NMR (400 MHz, CD_3OD, TMS), δ (ppm): 8.79 (s, 2H, εCH His), 7.33 (s, 2H, δCH His), 4.37 (dd, *J* = 6.2, 5.3 Hz, 2H, αCH His), 3.19 (dd, *J* = 15.2, 5.3 Hz, 1H, βCH His), 3.13 (dd, *J* = 15.2, 6.2 Hz, 1H, βCH His. **^{13}C NMR** (100 MHz, CD_3OD, TMS), δ (ppm): 169.1 (2 x CO); 135.3, 130.4, 118.9 (His); 55.2 (2 x αC); 29.3 (2 x βC). **MS (ESI)**: m/z 275.0 $(M+H)^+$.

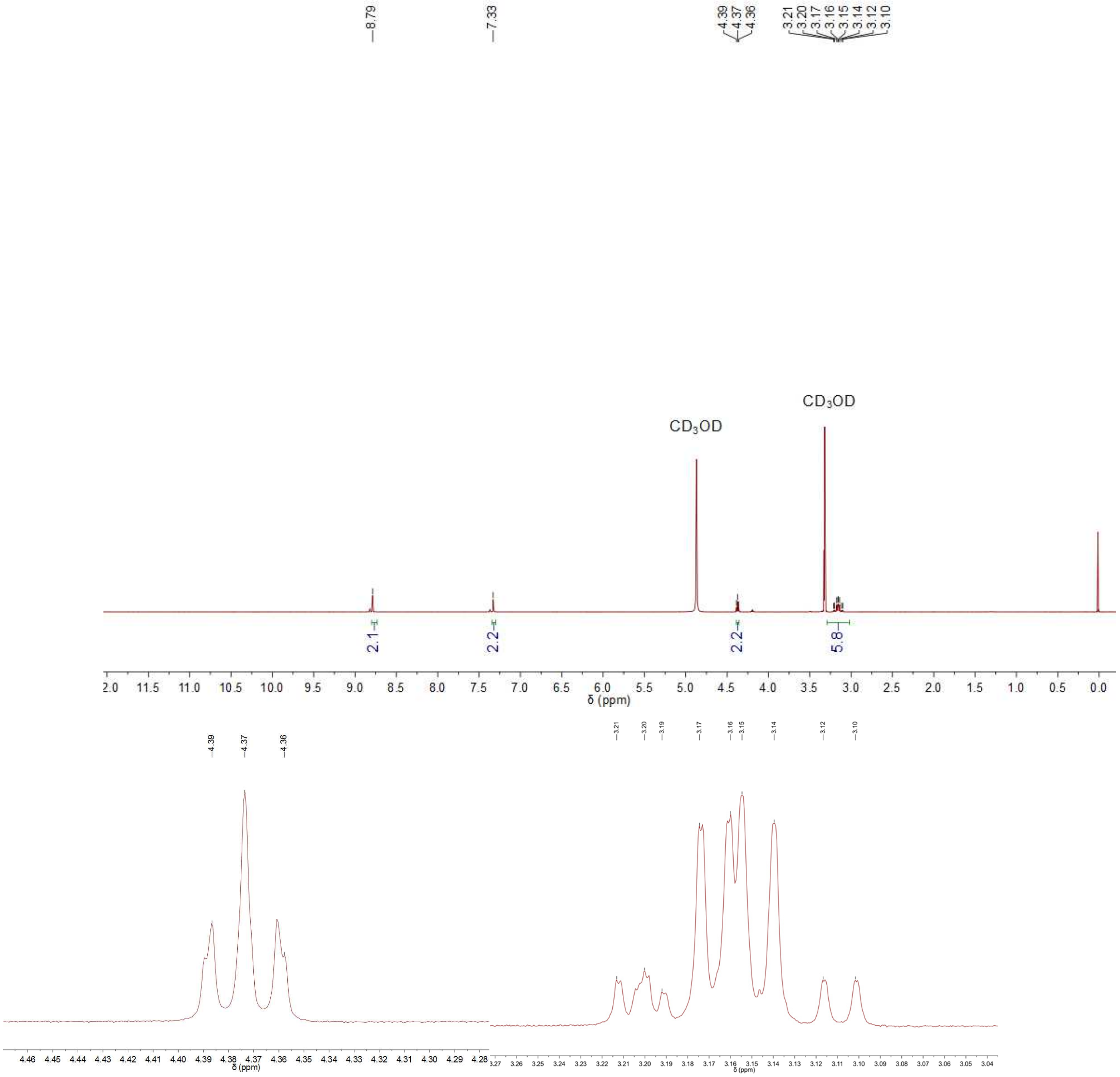

Figure S9. ^{1}H-NMR spectrum of DKP3 (full-view, top; detailed view, bottom).

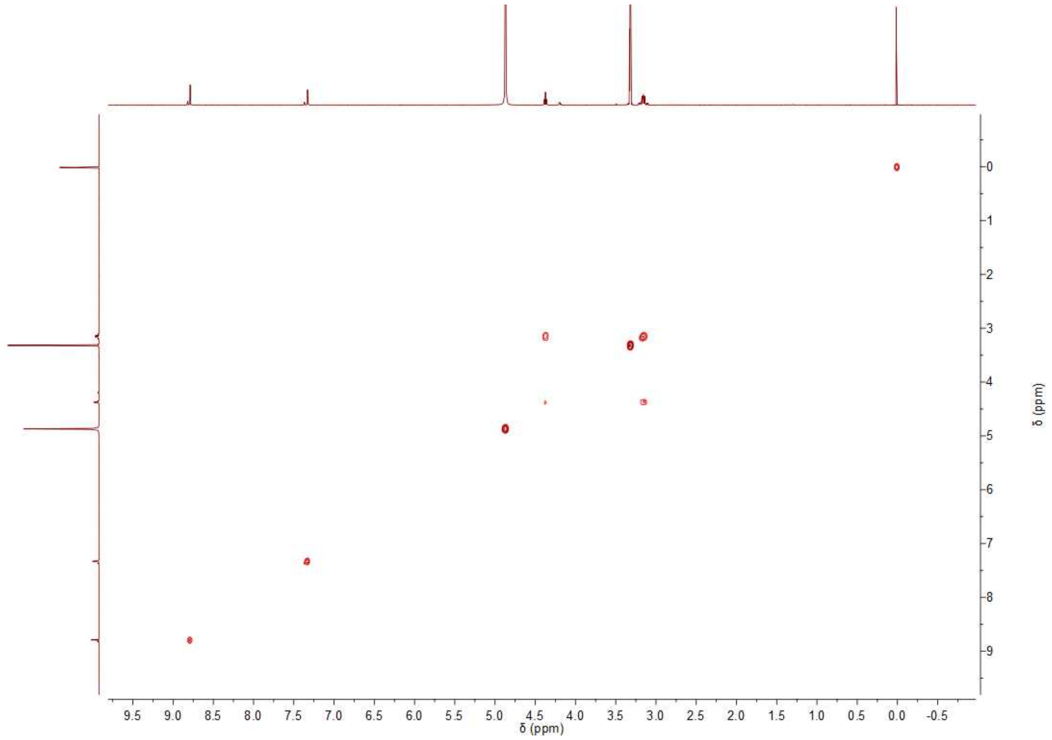

Figure S10. gCOSY 2D-NMR spectrum of DKP3.

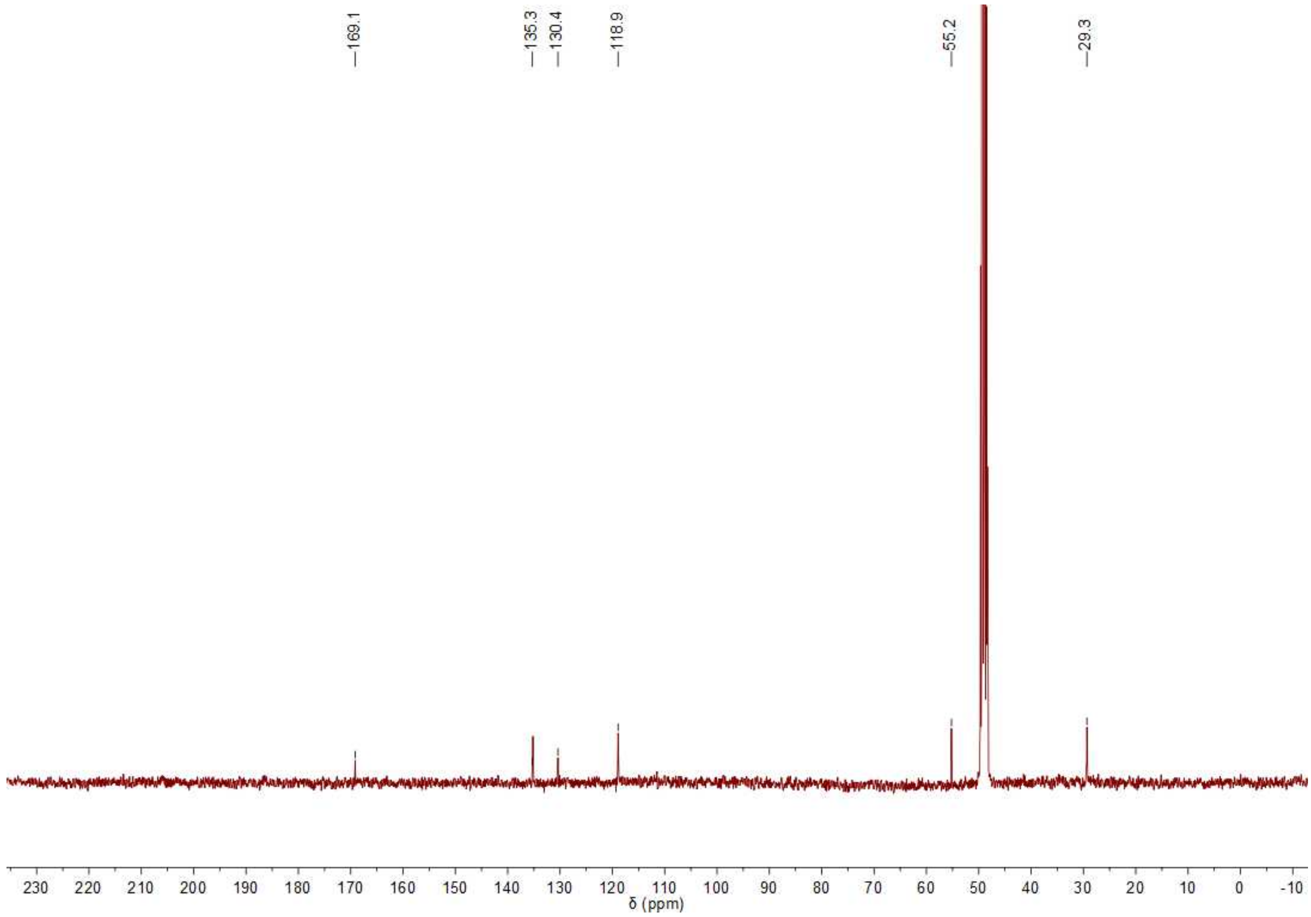

Figure S11. ^{13}C-NMR spectrum of DKP3.

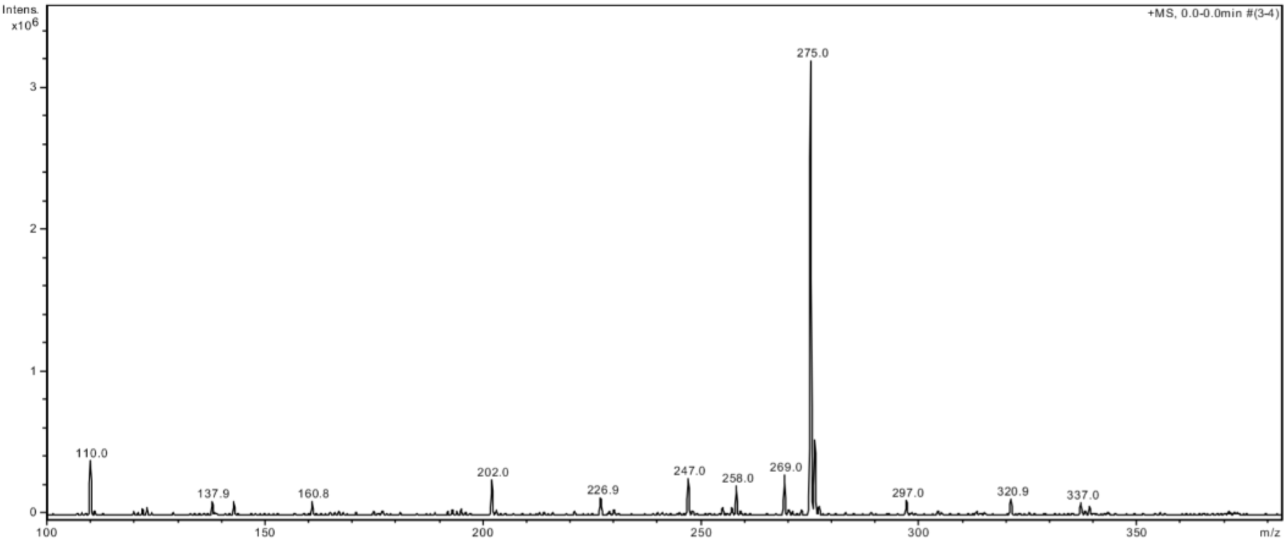

Figure S12. ESI-MS spectrum of DKP3 (positive ion mode).

4. Cyclo(His-Met) (DKP4) spectroscopic data

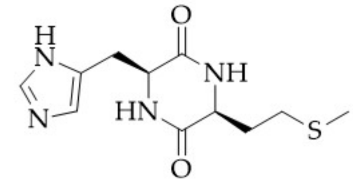

Cyclo(His-Met)
DKP4

^{1}**H NMR** (400 MHz, CD_3OD, TMS), δ (ppm): 8.29 (s, 1H, NH His), 8.04 (s, εCH His), 7.06 (s, 1H, δCH His), 4.52 (dd, *J* = 6.8, 5.2 Hz, 1H, αCH His), 3.95 (dd, *J* = 6.8, 6.8 Hz, 1H, αCH Met), 3.21 (dd, *J* = 5.2, 5.6 Hz, 1H, βCH His), 3.13 (dd, *J* = 6.8, 5.6 Hz, 1H, βCH His), 2.61 (dd, *J* = 7.6, 7.6 Hz, 2H, γCH_2 Met), 2.21 – 2.00 (m, 2H, βCH_2 Met), 2.11 (s, 3H, CH_3 Met). ^{13}**C NMR** (100 MHz, CD_3OD, TMS), δ (ppm): 169.7, 169.2 (2 x CO); 136.2, 132.7, 119.8 (His); 56.5, 54.9 (2 x αC); 34.5, 33.1 (2 x βC); 29.8 (ɣC Met); 15.0 (δC Met). **MS (ESI):** m/z 269.0 $(M+H)^+$.

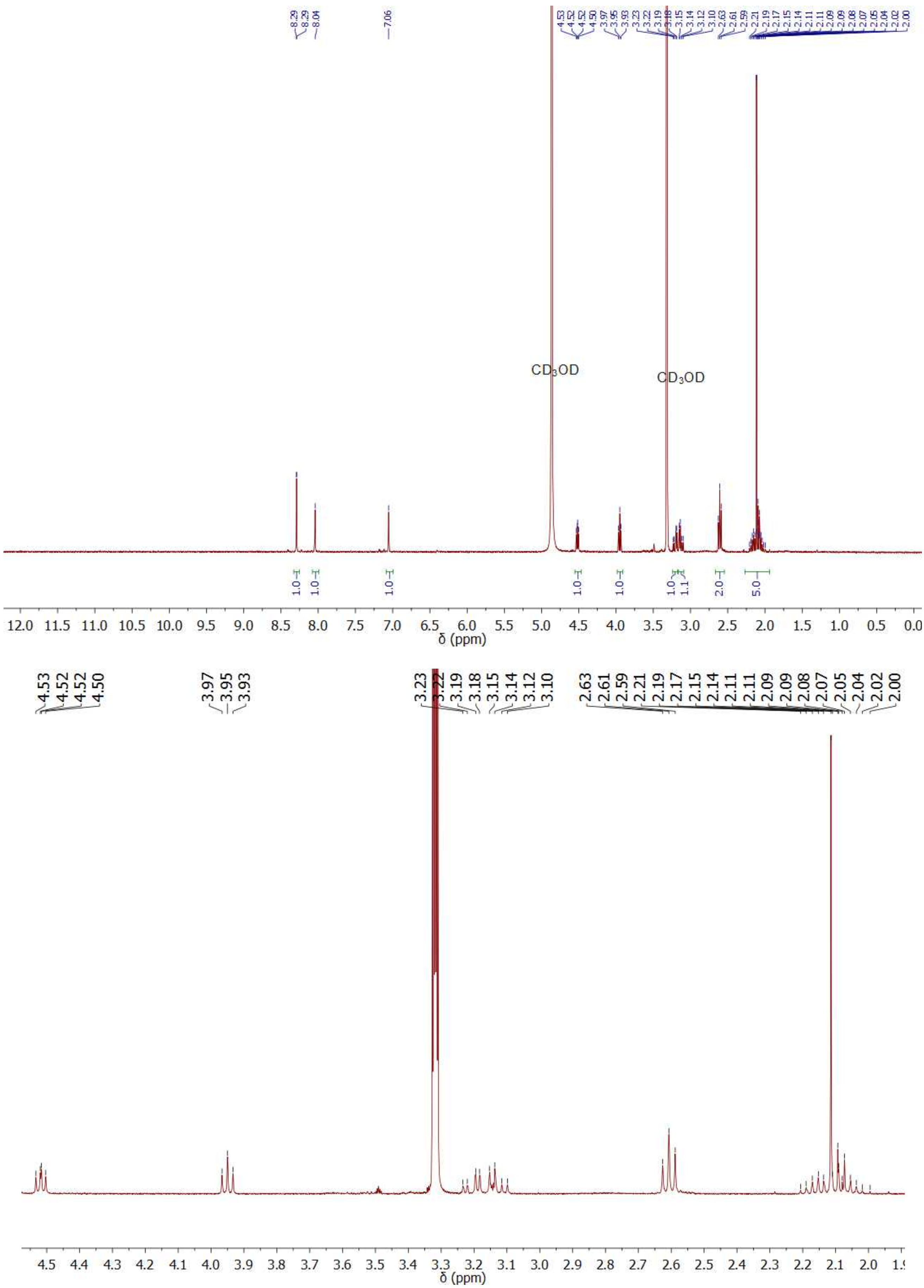

Figure S13. ^{1}H-NMR spectrum of DKP4 (full-view, top; detailed-view, bottom).

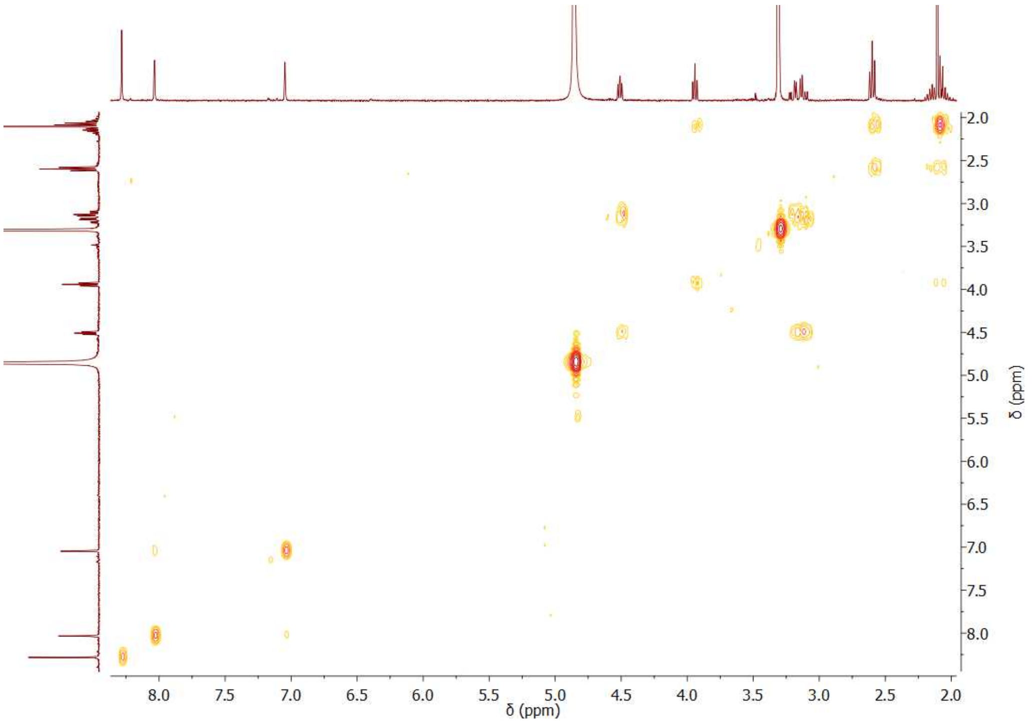

Figure S14. gCOSY 2D-NMR spectrum of DKP4.

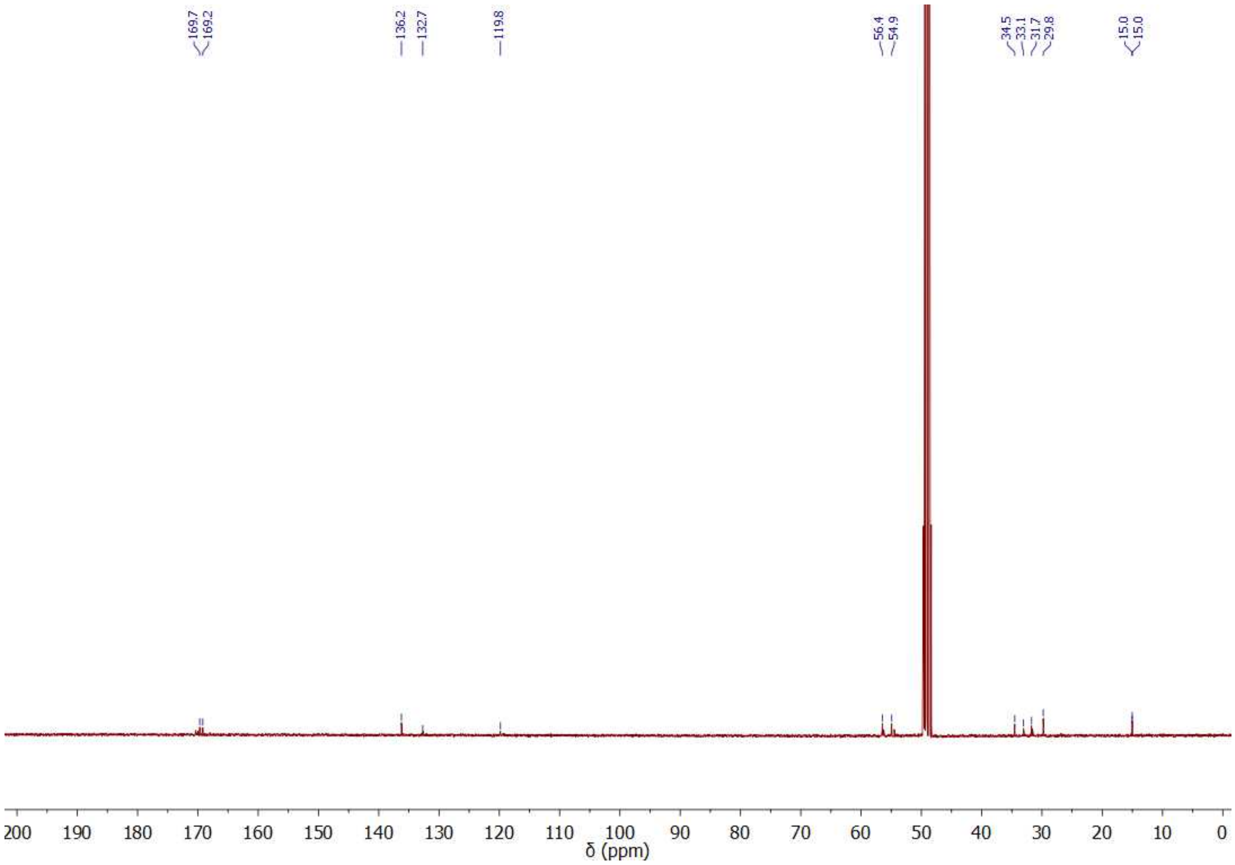

Figure S15. ^{13}C-NMR spectrum of DKP4.

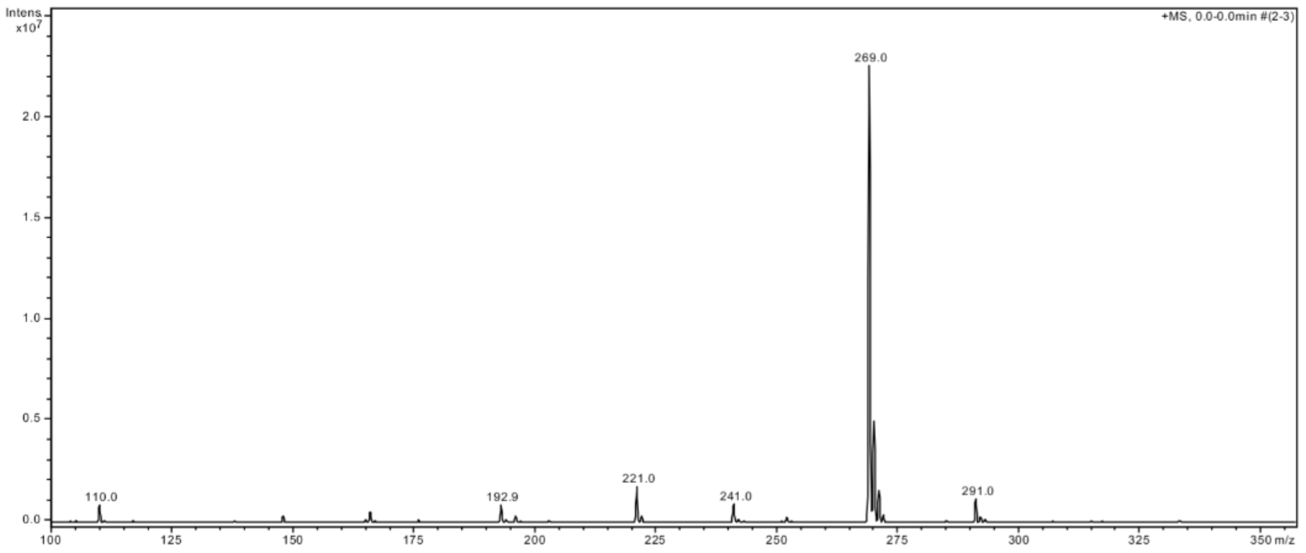

Figure S16. ESI-MS spectrum of DKP4 (positive ion mode).

5. Cyclo(His-Pro) (DKP5) spectroscopic data

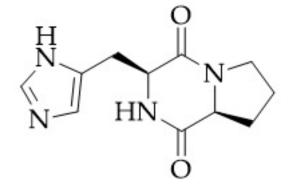

Cyclo(His-Pro)
DKP5

^{1}H NMR (400 MHz, CD_3OD, TMS), δ (ppm): 7.62 (s, εCH His), 6.93 (s, 1H, δCH His), 4.41 – 4.37 (m, 1H, αCH His), 4.26 – 4.21 (m, αCH Pro), 3.61 – 3.48 (m, 2H, δCH_2 Pro), 3.25 (dd, *J* = 15.2, 4.4 Hz, 1H, βCH His), 3.03 (dd, *J* = 15.2, 7.0 Hz, 1H, βCH His), 2.34 – 2.23 (m, 2H, βCH Pro), 2.04 – 1.80 (m, 3H, βCH, γCH_2 Pro). **^{13}C NMR** (100 MHz, CD_3OD, TMS), δ (ppm): 171.9, 167.5 (2 x CO); 136.4, 111.2 (His); 60.3, 56.5 (2 x αC); 46.4 (δC Pro); 29.3 (βC Pro), 23.4 (γC Pro). **MS (ESI):** m/z 235.0 $(M+H)^+$, 257.0 $(M + Na)^+$.

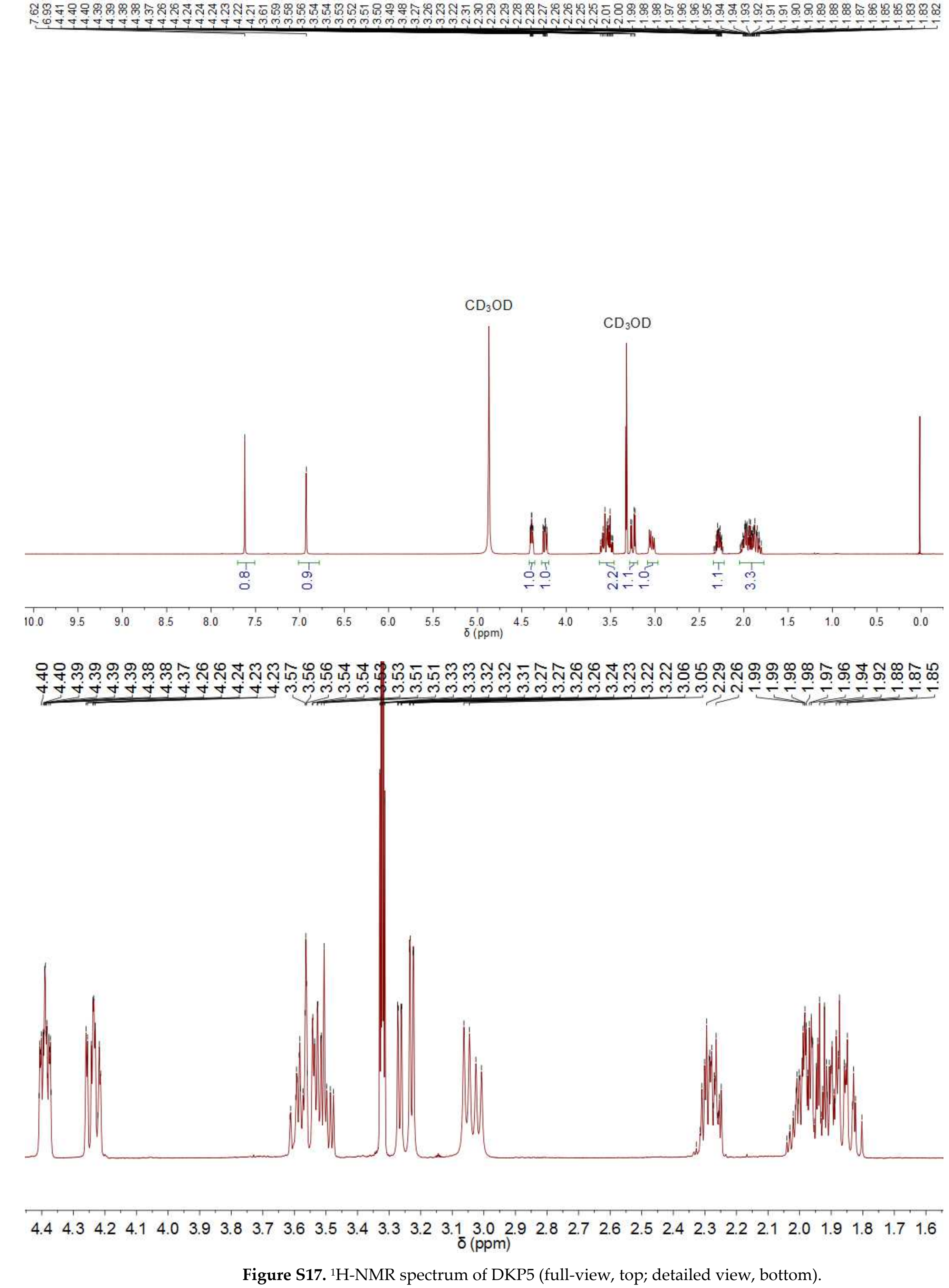

Figure S17. ^{1}H-NMR spectrum of DKP5 (full-view, top; detailed view, bottom).

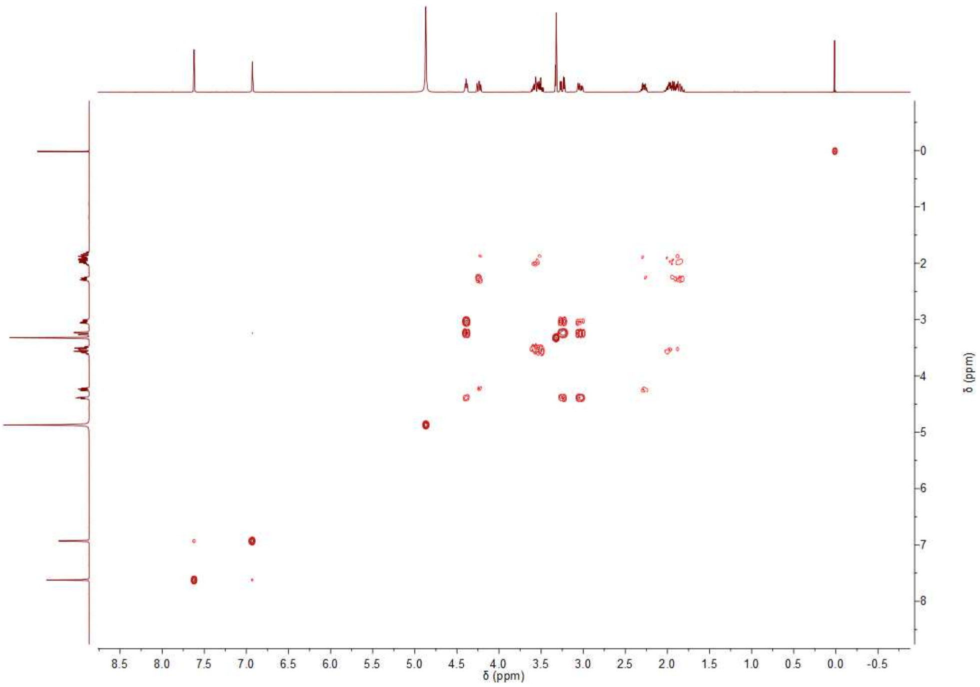

Figure S18. gCOSY 2D-NMR spectrum of DKP5.

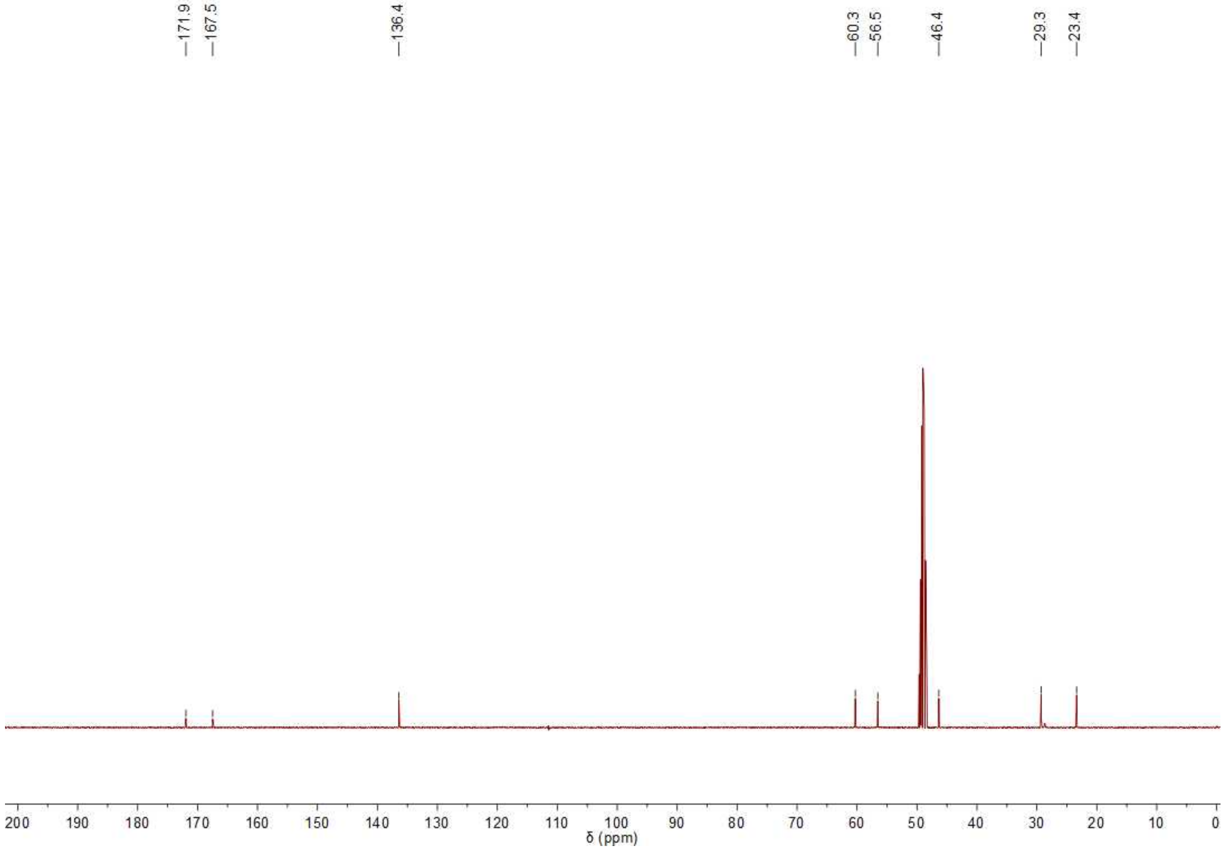

Figure S19. ^{13}C-NMR spectrum of DKP5.

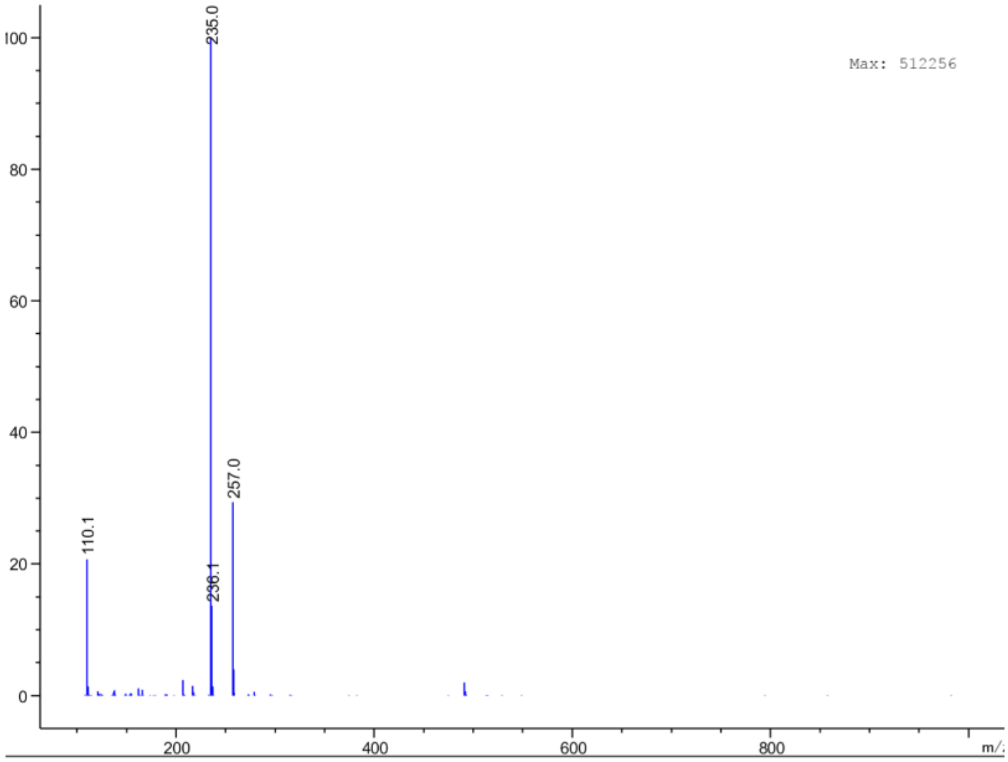

Figure S20. ESI-MS spectrum of DKP5 (positive ion mode).

6. Single-crystal X-ray diffraction

DKP1 (CCDC 2203140) and DKP2 (CCDC 2203142).

Crystals of DKP1 (CCDC 2203140) and DKP2 (CCDC 2203142) were mounted on the diffractometer at the synchrotron Elettra, Trieste (Italy), beamline XRD1 and measured at 100 K. Data collection were performed using synchrotron radiation (λ = 0.7000 Å) with the rotating crystal method (0.5°/image) for a total of 720 images. Data indexing were performed using MOSFLM,[1] while space groups were determined using POINTLESS.[2] The software AIMLESS[3] was used for scaling the data. The structures were solved using the software SHELXT[4] and refined through full matrix least-squares based on F^2 using the programs SHELXL[5] and OLEX2[6] as a GUI.

Non-hydrogen atoms were refined anisotropically, whereas hydrogen atoms were geometrically positioned and included in structure factor calculations but not refined, with the exception of the hydrogen attached to the nitrogen atoms in the DKP2 crystal structure, which were localized from the difference Fourier density maps and refined.

ORTEP diagrams (Figure S21) were drawn using OLEX2. In Table S1 are reported relevant the crystallographic data.

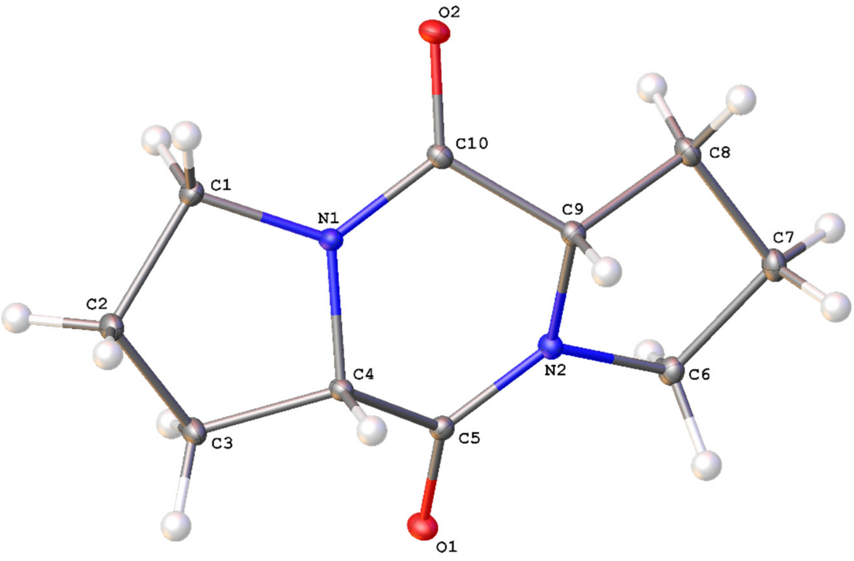
O2
C10
C8
C1
N1
C9
C7
C2
N2
C4
C6
C5
C3
O1

(a)

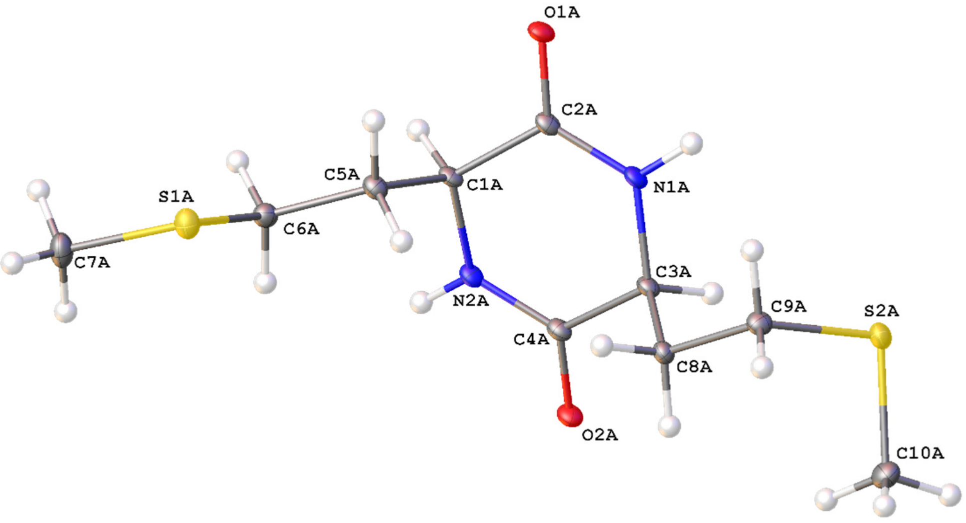
O1A
C2A
C5A
C1A
N1A
S1A
C6A
C7A
C3A
N2A
C9A
S2A
C4A
C8A
O2A
C10A

(b)

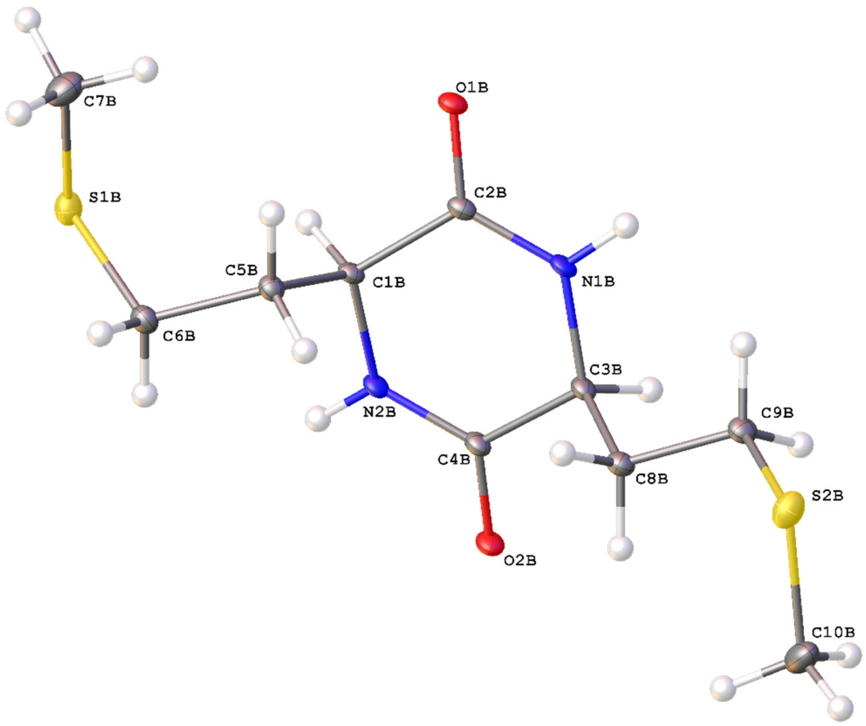

(c)

Figure S21. ORTEP diagrams of (a) DKP1 (CCDC 2203140) and (b, c) DKP2 (CCDC 2203142). For DKP2, both independent molecules in the asymmetric unit are reported. Atom types: C grey, H white, O red, N blue, S yellow.

Table S1. Relevant crystallographic data for the crystal structures DKP1 (CCDC 2203140) and DKP2 (CCDC 2203142).

	**DKP1 (CCDC 2203140)**	**DKP2 (CCDC 2203142)**
T (K)	100	100
Formula	$C_{10}H_{14}N_2O_2$	$C_{10}H_{18}N_2O_2S_2$
Formula weight	194.23	262.38
System	orthorhombic	monoclinic
Space group	$P2_12_12_1$	$P2_1$
a (Å)	5.7360(12)	6.0932(5)
b (Å)	9.1010(18)	18.5089(11)
c (Å)	17.771(4)	11.4749(8)
α (°)	90	90
β (°)	90	94.338(5)
γ (°)	90	90
V (Å^3)	927.7(3)	1290.41(15)
Z	4	4
D_x (g cm^{-3})	1.391	1.351
λ (Å)	0.70000	0.70000
μ (mm^{-1})	0.095	0.386
F_{000}	416.0	560.0
R1 (I > 2σI)	0.0353(2686)	0.0571(6596)
$_wR_2$	0.0916(2724)	0.1755(7068)
N. of param.	91	309
GooF	1.045	1.082
ρ_{min}, ρ_{max} (eÅ^{-3})	-0.41, 0.42	-0.63, 0.63
Restraints	/	/

[1] T. G. G. Battye, L. Kontogiannis, O. Johnson, H. R. Powell and A. G. W. Leslie, *Acta Crystallogr., Sect. D*, 2011, **67**, 271−281.

[2] P. R. Evans, *Acta Crystallogr., Sect. D*, 2006, **62**, 72−82.

[3] P. R. Evans and G. N. Murshudov, *Acta Crystallogr., Sect. D*, 2013, **69**, 1204−1014.

[4] Sheldrick, G. M., *Acta Crystallogr., Sect. A*, 2015, **71**, 3−8.

[5] Sheldrick, G. M., *Acta Crystallogr., Sect. C*, 2015, **71**, 3−8.

[6] O. V. Dolomanov, L. J. Bourhis, R. J. Gildea, J. A. K. Howard and H. Puschmann, *J. Appl. Cryst.*, 2009, **42**, 339−341.

7. Skin absorption data

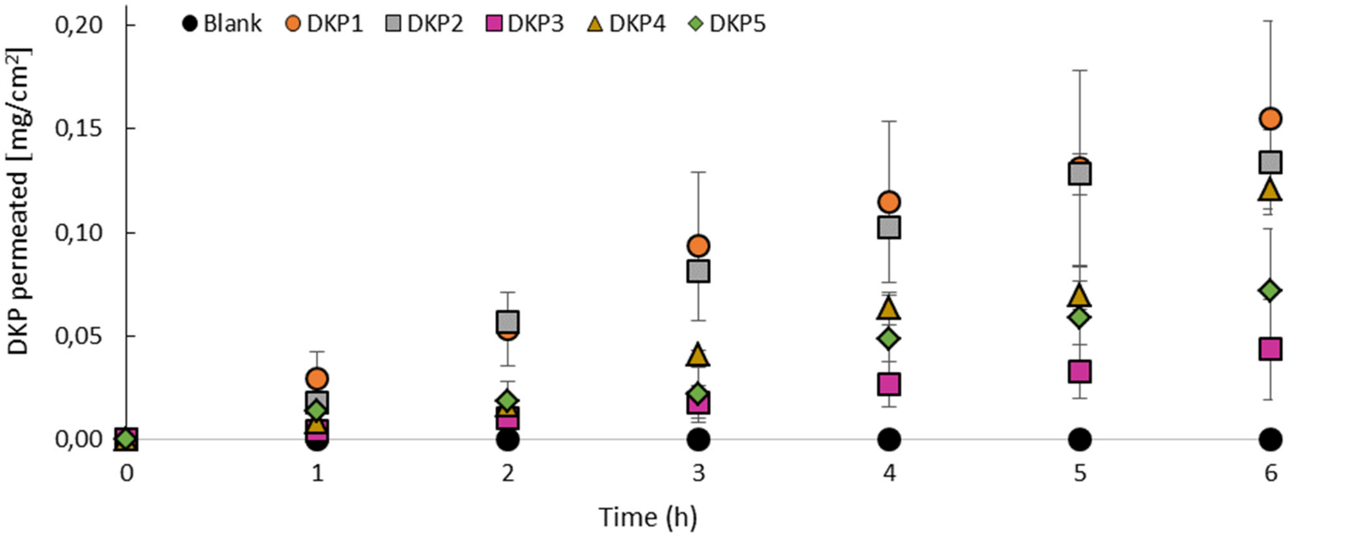

Figure S22. DKP concentrations (mg/cm^2) that permeated in the receptor medium at specific extraction times. Values are expressed as mean ± SD (n=6).

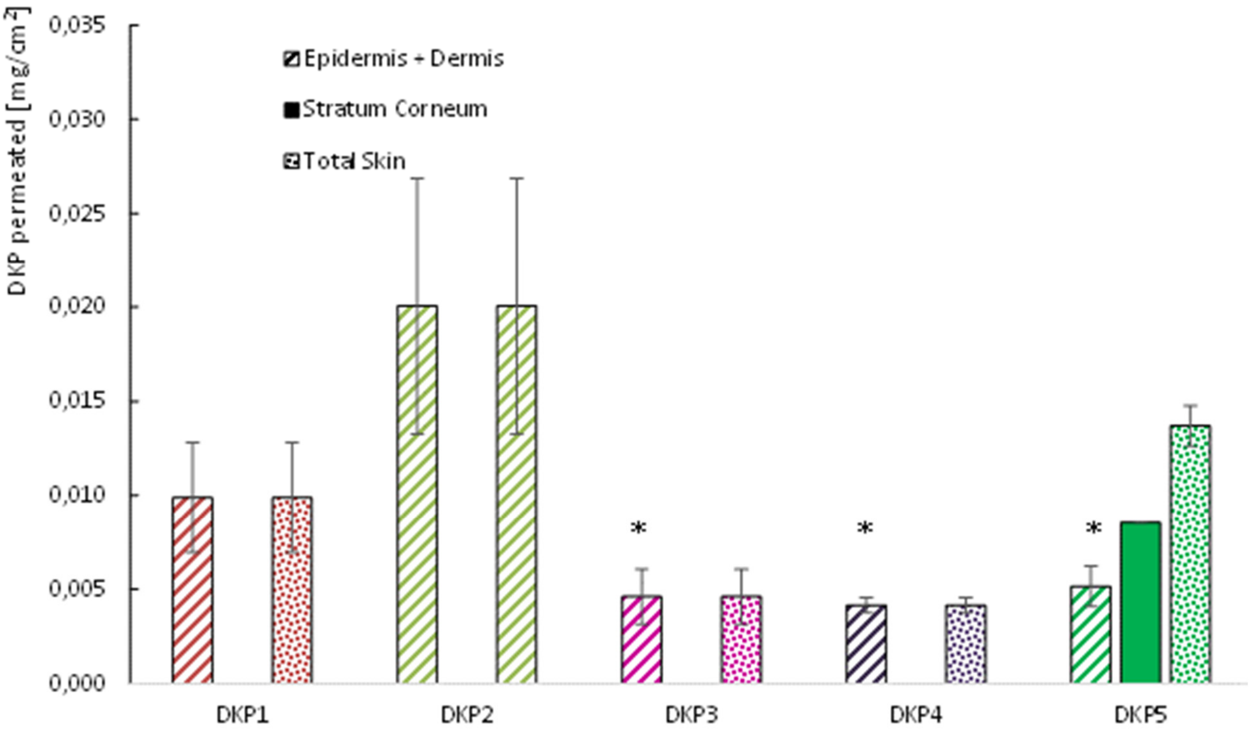

Figure S23. DKP concentrations found in skin layers after 6 h exposure. Applied dose was 0.53 mg/cm^2. Data are given as mean ± SD (n=6). Asterisk (*) indicates statistically significant ($p<0.05$).

Table S2. Statistical analysis of skin absorption data. p values are shown in the table as derived from the t test applied to data shown in figure S23.

T TEST	DC 6h	SKIN (E+D)	SKIN (SC)	RF 6h
**DKP1/DKP2**	0.25	0.08	n.a.	0.49
**DKP1/DKP3**	0.01	0.05	n.a.	0.02
**DKP1/DKP4**	0.06	0.03	n.a.	0.28
**DKP1/DKP5**	0.21	0.06	0.00	0.06
**DKP2/DKP3**	0.00	0.02	n.a.	0.01
**DKP2/DKP4**	0.00	0.02	n.a.	0.25
**DKP2/DKP5**	0.77	0.02	0.00	0.03
**DKP3/DKP4**	0.00	0.63	n.a.	0.01
**DKP3/DKP5**	0.00	0.63	0.00	0.27
**DKP4/DKP5**	0.00	0.20	0.00	0.05
